# BICEPP: an example-based statistical text mining method for predicting the binary characteristics of drugs

**DOI:** 10.1186/1471-2105-12-112

**Published:** 2011-04-21

**Authors:** Frank PY Lin, Stephen Anthony, Thomas M Polasek, Guy Tsafnat, Matthew P Doogue

**Affiliations:** 1 Centre for Health Informatics, The University of New South Wales, Sydney, Australia; 2 Department of Clinical Pharmacology, Flinders University, Adelaide, Australia; 3 Flinders Medical Centre, Adelaide, Australia

**Keywords:** data mining, artificial intelligence, drug toxicity, adverse drug reaction reporting systems, cytochromes P450

## Abstract

**Background:**

The identification of drug characteristics is a clinically important task, but it requires much expert knowledge and consumes substantial resources. We have developed a statistical text-mining approach (BInary Characteristics Extractor and biomedical Properties Predictor: BICEPP) to help experts screen drugs that may have important clinical characteristics of interest.

**Results:**

BICEPP first retrieves MEDLINE abstracts containing drug names, then selects tokens that best predict the list of drugs which represents the characteristic of interest. Machine learning is then used to classify drugs using a document frequency-based measure. Evaluation experiments were performed to validate BICEPP's performance on 484 characteristics of 857 drugs, identified from the Australian Medicines Handbook (AMH) and the PharmacoKinetic Interaction Screening (PKIS) database. Stratified cross-validations revealed that BICEPP was able to classify drugs into all 20 major therapeutic classes (100%) and 157 (of 197) minor drug classes (80%) with areas under the receiver operating characteristic curve (AUC) > 0.80. Similarly, AUC > 0.80 could be obtained in the classification of 173 (of 238) adverse events (73%), up to 12 (of 15) groups of clinically significant cytochrome P450 enzyme (CYP) inducers or inhibitors (80%), and up to 11 (of 14) groups of narrow therapeutic index drugs (79%). Interestingly, it was observed that the keywords used to describe a drug characteristic were not necessarily the most predictive ones for the classification task.

**Conclusions:**

BICEPP has sufficient classification power to automatically distinguish a wide range of clinical properties of drugs. This may be used in pharmacovigilance applications to assist with rapid screening of large drug databases to identify important characteristics for further evaluation.

## Background

A frequent inquiry in biology and medicine is to ask whether a biomedical entity (e.g., a drug) and a characteristic (e.g., an adverse effect) are associated with each other. Such true-false relationships form the core of scientific hypotheses. As they are crucial to our interpretation of biomedical phenomena, considerable amount of manpower and resources are often spent on their discovery and assimilation. Field experts frequently conduct extensive literature reviews and database searches to examine the evidence of these relationships. Furthermore, this binary knowledge often presents ambiguity that further restricts the rate of discovery.

Computational text mining tools, the automated analysis of biomedical texts stored in electronic media, have been developed to assist clinical and basic scientists in matching characteristics with domain-specific biomedical entities. For example, several methods of *in silico *candidate gene prioritisation have been developed that use features derived from MEDLINE to help scientists test whether a gene is likely to be associated with a clinical disorder [1-12]. Text mining has also been applied to classify clinical properties of drugs for use in quantitative structure-activity relationship (QSAR) models to accelerate drug development [13]. Mining text in electronic databases has also been integrated in clinical research including the automated classification of aetiological factors of cancers [14] and to match candidate anti-neoplastic drugs with cancers prior to clinical trials [15]. In the systematic organisation of scientific knowledge, text mining methods have been shown to be equally effective compared to the manual curation of pharmacogenetic databases [16].

In this paper, we have extended the application of text-mining to the task of identifying binary drug characteristics. We have developed a novel method, the BInary Characteristics Extractor and biomedical Properties Predictor (BICEPP), to classify properties (characteristics) of drugs (scientific entities) and subsequently validated this approach on data collected from traditional analytical methods derived from the knowledge of field experts (a therapeutic drug reference and a drug interaction database). To demonstrate its applicability, we evaluated the performance of BICEPP on many drug characteristics, including therapeutic classes, adverse effects, and their potentials for pharmacokinetic drug-drug interactions. The practical aim of BICEPP is to perform systematic, rapid throughput screening to help editors of drug references to redirect skilled staff to the evaluation of the resulting leads. Furthermore, the text mining approach for predicting drug characteristics may help to identify obscure adverse drug events (ADR). Specifically, the analysis of biomedical literature may further augment the existing models for ADR identification which are frequently based on physicochemical properties of drugs with QSAR modelling [13,17].

A notable feature of our approach is that it predicts drug characteristics by only using a list of drug names as examples supplied by user. This approach is advantageous because a well-constructed query is needed when performing a manual search of literature database. Such an example-based approach permits BICEPP to operate under situations where the concept of characteristic is unclear, or when the exact search terms and ranking methods are difficult to ascertain. The key concept employed by BICEPP is based on the *frequency *of textual features within biomedical text corpus. One of the commonly-used measures in information retrieval (IR) tasks is *term frequency *(*tf*), often defined as the normalised number of occurrences of a term (e.g., a word) in a given length of text. *Tf*-based measures have been applied to MEDLINE databases to predict gene-disease relationships [1,4,6]. In the search for genes related to rheumatoid arthritis, the term frequency-inverse document frequency *(tf-idf) *of biomedical concepts derived from MEDLINE abstract data was integrated with meta-analysis of genome-wide association studies to successfully identify novel single nucleotide polymorphisms (SNPs) associated with increased disease risk [12,18]. *Tf-idf*-based similarity comparisons have also been applied to assist with the manual classification of protein domain databases with high accuracy [19]. Hence, based on the generalisability demonstrated in other studies, it was justifiable to use a frequency-based approach to perform binary predictions and classification. In this paper, we explored the use of frequency-based methods to identify discriminative textual features for classifying drug characteristics.

## Methods

### Drug lists and drug characteristics studied in this paper

Two data sources were used in the training and evaluation of BICEPP models: 1) We manually extracted the generic names of all 857 drugs and 455 drug characteristics (238 adverse effects, 20 major therapeutic classes, and 197 minor therapeutic classes including indications) listed in the Australian Medicines Handbook 2009 (AMH) [20]. AMH is a clinical resource compiled by experts and is used for prescribing decision support. AMH was selected because its information content is reasonably complete, thus the assignment of binary classes to a drug (e.g., whether a drug may cause to an adverse event) can be performed without ambiguity. Only the generic names of drugs registered in Australia as listed in the AMH 2009 were used for the analysis. 2) The characteristics of pharmacokinetic drug-drug interaction were extracted from the PharmacoKinetic Interaction Screening (PKIS) database [21], including 15 "perpetrator" classes (capable of significantly altering the concentration of another drug) and 14 classes of narrow therapeutic index drugs. The number of characteristics in each group is listed in Table 1. Each of the 857 drugs was manually labelled positive (has the characteristic) or negative (does not have the characteristic) according to AMH and PKIS. The datasets are supplied as an additional file (see Additional File 1).

**Table 1:** The drug characteristics and their data sources

Data source	Characteristic category	Examples	Number of characteristics	Median number of drugs associated with the characteristic
Australian Medicines Handbook (AMH)	Major drug classes	Gastrointestinal drugs, Anti-infectives, Cardiovascular drugs	20	37
	Minor drug classes	5HT3 antagonists, Benzodiazepines, Carbapenems, Platinum compounds, Taxanes	197	3
	Adverse events	Anorexia, EPSE, Hyperuricaemia, Increased liver enzymes, Nephrotoxicity	238	22
Pharmacokinetic Interaction Screening tool (PKIS)	Perpetrators*	CYP1A2 inducers, CYP3A inducers CYP3A inhibitors (moderate), CYP3A inhibitors (strong)	15	5
	Narrow therapeutic index drugs	Alkylating agents, Anticonvulsants, Immunosuppresants,	14	5.5

Total			484	

Abbreviations: CYP = cytochromes P450. EPSE = extra-pyramidal side-effects. Note: *) Perpetrators: perpetrators are drugs that are capable of altering the concentration of another drug with ≥ 2-fold change via the hepatic CYP enzyme system.

### Estimating the conditional document frequency (CDF) of a token by MEDLINE search

The PubMed database (2009 baseline, accessed April 2009) was searched using the names of each AMH-listed drug as a query to retrieve all abstracts containing the drug name. We used words and hyphenated expressions as tokens: each abstract was transformed into a list of tokens delimited by white space, comma, and semicolon into an unordered set (bag of tokens), and common and stop words (e.g., "the", "and", "an", and "it") were not included in the set. Word stemming was not employed, case was ignored, and numerical data and digits were retained as part of the token. The *conditional document frequency *(CDF) of a token was defined as the number of abstracts normalised against the total number of abstracts retrieved from MEDLINE using keywords comprised of drug name *d*, such that:

where *cdf_w,d _*is the CDF of token *w *and drug *d*, and *N*(**q**) is the number of abstracts retrieved from MEDLINE containing the query comprised of tokens **q**.

### Eliminating rare and common tokens

To improve the efficiency in finding predictive tokens, we applied two heuristics to reduce the number of features in the search space. We removed rare tokens that occur in less than two abstracts in all drugs. Common tokens were eliminated if their CDFs were correlated linearly with *N*(*d*) (cut-off: *r^2 ^*> 0.33, where *r *is the Pearson's correlation coefficient).

### Selecting the most predictive tokens for classification

Tokens that best predict a drug characteristic, i.e., those with higher CDF in the positive examples (drug examples representing the characteristic of interest), were preferentially selected for subsequent classification: for each token *w *and the set of drugs representing the characteristic **C**, we defined both the true and false positive rates [TPR_*w*,**C**_(*t*) and FPR_*w*,**C**_(*t*)] of a token *w *with respect to a characteristic **C **as:

for all drugs *d *∈ **D **= {*d*_1_, *d*_2_,..., *d*_857_} listed in the AMH, where *t *is an arbitrary threshold score bound by 0 and 1, **C **is contained in **D**, and *n*(*d *∈ **C**) and *n*(*d *∉ **C**) denote the numbers of abstract containing drug name *d *and in/not in **C **respectively.

The corresponding area under ROC curve (AUC) over all thresholds is defined as:

where 1 = *t*_0 _>*t*_1 _>*t*_2 _> ... >*t*_N _= -0 representing all possible thresholds of CDFs across the list of drugs **D**. In cross-validation experiments, this feature selection process was conducted independently on the training folds instead of all data to avoid contamination of information from the test folds.

### Using CDFs and machine learning algorithms to predict drug characteristics

We selected four machine learning algorithms for this classification task: naive Bayes (NB), *k*-nearest neighbour (IBk) with inverse distance weighing with *k *determined by cross-validations, and support vector machines (SVM) with linear (SVM/Linear) and radial basis function kernels (SVM/RBF). The CDFs of the most discriminative tokens (as measured by highest AUCs) were used to train machine learning models. To obtain consistent experimental results across a wide range of characteristics, we elected to use a fixed number of features (top-20 most discriminative tokens) for comparative evaluations (see Additional File 2 for further explanation). Waikato Environment of Knowledge Analysis [22] was used for constructing classifiers and making predictions.

### Estimating BICEPP's performance by stratified cross-validation

**Figure 1:**
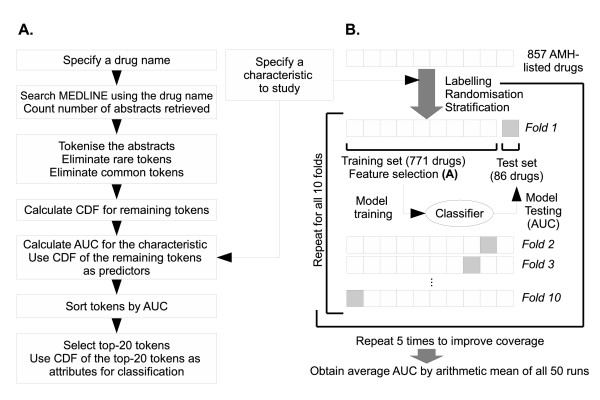
**The workflow of BICEPP and the evaluation procedure**. A. The procedures of feature derivation and feature selection. The features for the inputs of machine learning classifiers are the CDF of 20-most predictive tokens. The CDF of a token, given a drug, is defined as the proportion of abstracts containing the token within the list of abstracts retrieved by using the drug name as query to search MEDLINE. B. Cross-validation was performed to estimate the generalisation performance of BICEPP. The feature selection described in (A) was performed on the training set (which contains *k*-1 folds of data) and machine learning models were built to predict test set data. This figure illustrates the 5 × stratified up-to-10-fold cross-validation procedure used throughout the evaluation experiments in this paper. Abbreviations: AMH: Australian Medicines Handbook; AWT: abstract with title; AUC: area under ROC curve; CDF: conditional document frequency using the drug name as query to search MEDLINE; ROC: receiver operating characteristics;

The generalised classification performance was estimated by stratified cross-validations: 1) The list of drugs was first *labelled *either a positive or negative class according to whether they are associated with the characteristic of interest. 2) *Randomisation *was subsequently performed to ensure homogeneity across different folds of training data. 3) The list of drugs was subsequently *stratified *to ensure that each fold contains roughly the same number of positive and negative drug examples. For a characteristic with 10 or more positive examples, five cycles of stratified 10-fold cross-validation were performed. For characteristics with < 10 positive examples, the number of folds was reduced to match the number of positive examples (*k*-fold cross-validation, 1 <*k*< 10) to avoid constructing a test set without positive examples. 4) The AUCs obtained from each run of prediction were averaged by an arithmetic mean. To examine whether one algorithm outperformed another, the number of characteristics with AUCs better than 0.80, 0.90, or 0.95 were counted for each classifier. The overall experimental procedure and the workflow of BICEPP are illustrated in Figure 1.

### Comparative evaluation with other IR methods for generating features for drug characteristics prediction

Four additional evaluations were performed to assess whether other commonly employed IR techniques may improve the performance of *cdf*-only predictions:

#### 1) Conditional term frequency

As a variation of *cdf*, the conditional term frequency (*ctf*) of a drug *d*, defined as the number of times a token appears in abstracts containing drug name *d *across all MEDLINE abstracts, was calculated for each drug such that:

where *n_i_*(*w*) is the number of tokens matching token *w *in the *i-*th document containing drug name *d *in MEDLINE. The common and rare tokens were eliminated using the identical procedure described above.

#### 2) Conditional term frequency-inverse conditional document frequency (ctf-icdf)

This measure is analogous to *tf-idf *with the difference that the document corpus was restricted only to the abstracts containing drug name *d*:

The global term and document frequencies of a token (*tf *and *df*, respectively defined as the number of tokens and documents present in MEDLINE *independent of *the co-occurrence of the drug name *d *in the abstracts) were not evaluated because both *tf *and *df *are constants hence are identical across all drugs. Stemming was not employed in both analyses (*ctf *and *ctf-icdf*) to standardise the comparisons with the CDF experiment.

#### 3) Application of stemming algorithm

Linguistically related tokens were grouped by using the stemming algorithm developed by Porter [23]. The effect of stemming on BICEPP performance was evaluated by using the CDF of each stemmed token for making predictions.

#### 4) Using drug synonyms to search the MEDLINE database

The use of drug synonyms can improve the recall of overall document retrieval, but its effect on the prediction of drug characteristics is unknown. To evaluate whether the incorporation of drug synonyms could have improved the predictive accuracy, we included the corresponding trade names listed in the Schedule of the Australian Pharmaceutical Benefit Scheme [24] to perform MEDLINE search. The trade names were pre-processed to remove the strength and formulation suffixes. For example, "Zydol SR 100" and "Zydol SR 200" were both reduced to "Zydol"; "Zofran" and "Zofran syrup" were both truncated to "Zofran". The CDF of each token was calculated and used for comparative evaluation of predictive performance.

## Results

### Feature selection process

A total of 1,814,157 abstracts were returned by searching all 857 AMH drug names against MEDLINE. Overall, a median of 890 abstracts was retrieved for a given drug (inter-quartile range, IQR: 275-2,446; maximum 198,950, "calcium"), with a median of 11,717 tokens per drug (IQR: 5,873-21,542). A median of 1,220 tokens (IQR: 515-1,489) was retained following the removal of rare and common tokens. On average, 86% of all tokens were eliminated prior to the feature ranking process. An example of eliminated tokens is shown in Table 2. The remaining tokens were ranked by the predictive power as measured by AUC. For 160 (33%) characteristics, there was at least one token that perfectly predicted the characteristic itself (AUC = 1). For example, the adverse effect of "sinus tachycardia" can be perfectly discriminated by the CDF of the token "tricyclic", whereas the CDFs of tokens "sexual", "naion", "pde5a1", and "self-confidence" can perfectly predict whether a drug is a type 5 phosphodiesterase inhibitor. Only 22 (4.5%) characteristics had the most discriminative token with AUC < 0.70 across all 857 drugs.

**Table 2:** Examples of tokens eliminated and retained during the feature selection process on drug "warfarin"

*r^2^*	Examples of tokens
≈ 1 (perfect correlation)	only, when, however, well, another, same, results, other, observed, possible, different, since, even, could, though, occurring, therefore, high, although, also, both, so, result, appeared
≈ 0.90	restricted, controls, implicated, followed, diverse, stable, display, rate, plays, indicative, inhibit, typically, describe, excluded, terminal, excessive, largest, knowledge, employing, se
≈ 0.80	life, mature, loading, preincubation, problem, failure, binds, resolved, physiology, shock, signs, molecule, bind, elevations, chinese, usual, surface, aid, unit, accurate
≈ 0.70	intervention, stimulus, transition, closed, enable, bands, requiring, ester, nervous, sizes, electrophoresis, polymorphonuclear, aging, associations, accounts, practical, selective, choice, routine, attached
≈ 0.60	subset, undergoes, success, antagonist, artery, mr, depolarization, fields, suppression, precipitation, temperatures, records, mg2, adjustment, oxygen, picture, assembly, transcripts, encoded, organic
≈ 0.50	hydrogen, coated, glycol, antisense, coronary, adsorbed, histology, scan, formulation, foods, holding, resorption, gestational, filling, locus, memory, atrophy, ringer, prospectively, recruitment
≈ 0.40	diuretics, atrial, lysis, spinal, camp, bmax, vein, proteases, chelator, arachidonic, alzheimer, ascorbic, histamine, rhythm, ouabain, gas, preoperative, bladder, menopause, pertussis
≈ 0.33 (moderate correlation)	chromatographic, endothelin, relaxed, acceptable, stenosis, withdrawal, january, trypsin, oxidized, infiltration, forearm, et-1, enrolled, electrochemical, peroxidation, mothers, phosphodiesterase, cystic, compression, countries

*r *= Pearson's correlation coefficient

Examples of tokens retained by feature selection (in decreasing order of document frequencies)

Warfarin (8060), anticoagulation (2508), anticoagulant (1953), heparin (1699), thrombosis (1651), bleeding (1633), international (1324), venous (1238), aspirin (1231), fibrillation (1191), inr (1106), prothrombin (1035), thromboembolism (1017), anticoagulants (864), thromboembolic (860), coagulation (790), embolism (706), deep (698), prophylaxis (636), antithrombotic (606)

Examples of rare tokens eliminated by feature selection

vestige, bacteroides, ca-laurell, gd2, idaho, i475s, h2-blocker, depots, viic/viiam, left-hemispheric, p = .37, laboratory-developed, cardio, frames, thistle, thy1, homolog, videotapes, u-105665, five-years, cold-labeled, workups, fviiic

Examples of common tokens eliminated by feature selection

### Comparative evaluation of machine learning predictions using CDFs as features

**Figure 2:**
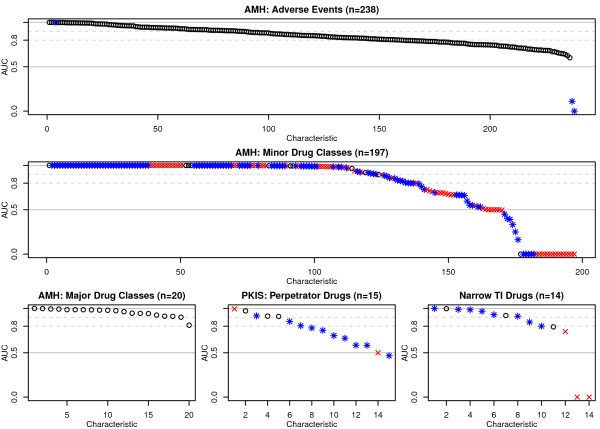
**The predictive performance of BICEPP (AUC) by drug characteristics**. The predictive power of BICEPP was evaluated by using stratified cross-validation experiments performed on each of the 484 drug characteristics listed in Table 1. In this figure, each data point denotes the best AUC (out of the 4 machine learning algorithms) evaluated on the dataset of drug characteristic that contains more than 10 positive examples (O), between 2--9 positive examples (*), and less than 2 positive examples (X) respectively. The dotted lines indicate AUCs of 0.8 and 0.9 respectively. Abbreviations: TI: therapeutic index.

By inspecting the cross-validation results across all categories, 20 (100%), 135 (69%), 159 (67%), 7 (47%), and 9 (64%) of the drug characteristics could be predicted with good cross-validation performances (AUC > 0.80), with the best performing algorithms, for categories of the AMH major therapeutic classes, minor therapeutic classes, adverse drug reactions, PKIS perpetrators, and PKIS narrow therapeutic index drug classes, respectively. In particular, BICEPP was very good at predicting major therapeutic classes (95% of drugs with AUC > 0.90) but less good at predicting drugs that may alter CYP-mediated metabolism (the perpetrators drugs, 33%). For the minor therapeutic classes (containing drug indications), 123 (62%) of the characteristics achieved AUC > 0.9 and the performance was less for the remaining datasets. The cross-validation results by characteristics and algorithms are summarised in Table 3 and Figure 2. The full results are also supplied as an additional file (see Additional File 3).

**Table 3:** The predictive performance of BICEPP by characteristics categories

Category	Best AUC	Algorithm				
		**NB**	**IBk**	**SVM/L**	**SVM/RBF**	***Best of 4***

AMH major classes	> 0.80	20 (100)	19 (95)	19 (95)	20 (100)	**20 (100)**
	> 0.90	15 (75)	16 (80)	16 (80)	16 (80)	19 (95)
	> 0.95	10 (50)	12 (60)	11 (55)	11 (55)	12 (60)
AMH minor classes	> 0.80	98 (50)	133 (68)	130 (66)	134 (68)	**135 (69)**
	> 0.90	86 (44)	121 (61)	120 (61)	117 (59)	123 (62)
	> 0.95*	73 (37)	114 (58)	102 (52)	106 (54)	114 (58)
AMH adverse events	> 0.80	134 (56)	145 (61)	114 (48)	119 (50)	**159 (67)**
	> 0.90	65 (27)	76 (32)	56 (24)	63 (26)	86 (36)
	> 0.95	30 (13)	38 (16)	30 (13)	35 (15)	41 (17)
PKIS perpetrator	> 0.80	3 (20)	7 (47)	3 (20)	4 (27)	**7 (47)**
	> 0.90	1 (7)	4 (27)	2 (13)	3 (20)	5 (33)
	> 0.95	0 (0)	2 (13)	2 (13)	2 (13)	2 (13)
Narrow therapeutic index drugs	> 0.80	8 (57)	9 (64)	8 (57)	8 (57)	**9 (64)**
	> 0.90	7 (50)	8 (57)	5 (36)	7 (50)	8 (57)
	> 0.95	3 (21)	5 (36)	3 (21)	2 (14)	5 (36)

*Overall*	> 0.80	263 (54)	313 (65)	274 (57)	285 (59)	**330 (68)**
	> 0.90	174 (36)	225 (46)	199 (41)	206 (43)	241 (50)
	> 0.95*	116 (24)	171 (35)	148 (31)	156 (32)	174 (36)

The numbers in this table indicate the number of characteristics (percentage) that achieved an AUC above the given threshold in stratified cross-validation evaluations. The performance is indicated by AUC and can be interpreted as good (> 0.80), very good (> 0.9), and excellent (> 0.95), respectively. Overall, 68% of drug characteristics can be predicted with good AUC (numbers in boldface) and 36% of characteristics can be predicted very accurately (AUC > 0.95) with at least one classifier. The last column (best of 4) shows how many characteristics achieved AUC above the given threshold by any of the four algorithms. Pearson's chi-square test was applied to examine the homogeneity between algorithms. *) indicate the statistically significant categories at α = 0.05 (analysed as 4 × 1 tables with 3 d.f.). However, no categories were statistically performance significant after adjusting for family-wise error rate using Bonferroni method (n = 18). Abbreviations: AE: adverse events; AMH: Australian Medicines Handbook; IBk: *k*-nearest neighbour algorithm; NB: Naive Bayes; SVM: support vector machine; SVM/L: linear SVM; SVM/RBF: support vector machine with radial basis function kernel. PKIS: PharmacoKinetic Interaction Screening database.

**Figure 3:**
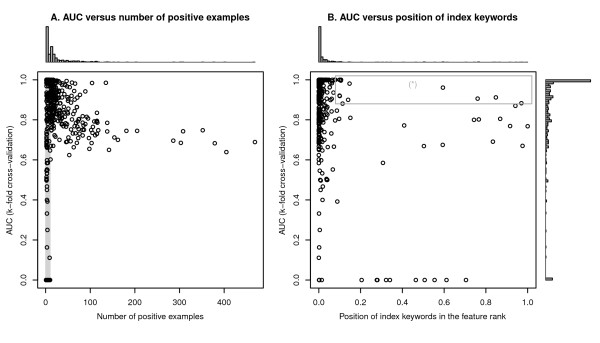
**The predictive performance versus number of positive examples and the position of index keywords**. This figure illustrates how the performance of BICEPP is related to the number of positive examples (A) and position of index keywords in the respective feature ranks (B). Each data point represents the best AUC (out of the 4 machine learning algorithms studied in this paper) performed on one of the 484 drug characteristics listed in Table 1. As illustrated in the shaded area in Figure 3(A), the predictive performance of BICEPP had a higher variability in datasets with less than 10 positive examples. The boxed area (*) in Figure 3(B) represents a list of "surprising characteristics", whose predictive powers were high but the index keywords were not discriminative. The contents are listed in more detail in Table 3. Refer to the main text for details.

The number of positive examples in a characteristic was loosely and inversely associated with classifier performance (Spearman's *σ *= -0.49 for characteristics with ≥ 10 positive examples, Figure 3A). For example, "nausea" was listed in 469 out of 857 drugs but cross-validations only yielded a best AUC of 0.689. This is in contrast with datasets with fewer positive examples (for example, "myelosuppression" was only labelled in 37 out of 857 drugs but had an AUC of 0.974). As expected, for characteristics with < 10 positive examples, there was a considerably higher variations in classification performance. For characteristics with ≥ 10 positive examples, 269 out of 272 (98.9%) had AUCs with the lower boundary of 95% confidence interval above 0.5 (one-sided z-test; standard errors were estimated by using Hanley-McNeil method [25]), indicating that the vast majority of performance estimates were significantly better than chance.

Machine learning algorithms were found to perform differently in the drug classification tasks. IBk achieved the best cross-validation results in most (53%) characteristics. Similarly, SVMs with both linear and RBF kernels achieved best cross-validation results in 21% and 23% of all drug characteristics, respectively. One of the frequently-used algorithms in IR and document classification, naïve Bayes, achieved best classification power in 52 (11%) of all characteristics. However, the number of characteristics identified with good (AUC > 0.80), very good (> 0.90), and excellent (> 0.95) discriminatory powers was not significantly different between algorithms (Table 3).

### The keywords used to describe a drug characteristic are not necessarily the most predictive tokens

It was observed that the keywords used to describe drug characteristics [the *index keywords*, (IK), e.g., "myelosuppression" is the IK of the corresponding characteristic dataset] were not necessarily placed highly on the token rank as sorted by AUC. In 145 (30%) of 484 characteristics evaluated, the IKs were either very common or very rare and hence eliminated by the feature selection process. For the remaining 70% of characteristics, 212 (62.5%) have IKs placed within one percentile from the top of token rank (Figure 3B). There were, however, 34 (10%) characteristics that had their IKs outside the top 10-percentile. Several interesting observations were made on these characteristics where the IKs were very lowly-ranked (lower than the 10^th ^percentile) but still maintained a very good overall predictive power (AUC > 0.9). For example, the adverse effect of "cystitis" was associated with predictive tokens that are indicative of non-steroidal anti-inflammatory drugs (NSAIDs); the adverse effect "oesophagitis" was found to be associated with tokens connected to metastatic cancer and chemotherapy (e.g., "weekly", "metastatic", "survival"); drugs that can potentially cause thrombocytopenic purpura were found to be linked with high word frequencies in keywords used in cardiology such as "echocardiography", "ST-segment", and "ejection" (Table 4).

**Table 4:** Drug characteristics with poorly discriminative index keywords but achieved an overall good predictive performance

Category	Characteristic	Position of IK(s)	n(pos)	Best AUC*	Top-20 predictive tokens/words (AUC^†^)
AE	Cystitis	10.7 pct	13	1.000	nsaids, cyclooxygenase, nimesulide, meloxicam (0.999); nsaid, diclofenac, naproxen, antiinflammatory, non-steroidal (0.998); ibuprofen, anti-inflammatory, nonsteroidal (0.997); ketoprofen, antipyretic (0.996); indomethacin (0.993); osteoarthritis (0.991); pge2 (0.991), prostanoid (0.988), thromboxane (0.986), prostaglandin (0.985)
AE	Dyslipidaemia	14.0 pct	13	0.900	Aldosterone (0.95); acetazolamide, mineralocorticoid (0.94); deoxycorticosterone (0.93), pge2, indomethacin, hearing (0.88); spironolactone, mineralocorticoids, hyponatremia, renin, adh, ace (0.87); furosemide (0.86); insipidus, asthmatic, prostaglandins, fev1, pra, phenylbutazone (0.85)
AE	Migraine	76.0 pct	14	0.906	angiotensin (0.89), plasminogen, dbp, insulin (0.85); infarction, low-density, losartan (0.84); hormonal, brachial, run-in, fixed-dose (0.83); lipoprotein, valsartan, endothelium-dependent, renin (0.82); pravastatin, hba1c (0.81); angiotensinogen, chd, smoking (0.80)
AE	Oesophagitis	10.0 pct	19	0.919	Metastases (0.92); marrow (0.90); weekly (0.88); metastatic, antitumor (0.87); cancer (0.86); 3-year, toxicity, regimen, nadir, breast, metastasis (0.85); myeloma, survival, cancers, prostate (0.84); regimens, remission, cytotoxic, melphalan (0.83)
AE	Paralytic ileus	59.4 pct	12	0.961	amitriptyline (0.96); tricyclic (0.95); antidepressants, anticholinergic, antidepressant (0.94); neuroleptics, chlorpromazine, depressive, overdose (0.93); tca, serotonin, diazepam (0.92); intoxication (0.91); thioridazine, clonidine (0.90); psychotropic, affective, psychological, antinociceptive (0.89); constipation (0.87)
AE	Thrombocytopenic purpura	10.6 pct	11	1	infarction, ejection (0.95); intra-arterial (0.94); echocardiography (0.93); st-segment (0.93); echocardiographic (0.90); beta-blocking (0.89); beta-blocker (0.87); cardiology, diacetolol, bopindolol, bucindolol, beta-adrenoceptor-blocking, adp, beta-ars, beta1-selective, beta-adrenoblockers, cardioselectivity, atenolol, non-fatal (0.86)
MC	5HT3 antagonists	10.0 pct	4	1	5-ht3ra, 5-ht3ra/dexamethasone, granisetron, ondansetron, 1966-september, 5-ht3, tropisetron, 5-hydroxytryptamine3, anti-emetic (1); dolasetron, emetic, ramosetron, 5-ht3-receptor, cinv, 5ht3, emetogenic, type-3, emesis, setrons, pov (0.999)
MC	Antibacterials (ear)	14.7 pct	4	0.98	enrofloxacin, chloramphenicol, gentamycin (0.99); oxytetracycline, kanamycin, polymyxin, colistin, gentamicin, bacitracin, neomycin, povidone-iodine, fusidic, streptomycin, bacterial, septicemia, swabs (0.98); anaerobic, peru, tetracycline, aminoglycoside (0.97)

*) Refers to the best area under the ROC curve achieved by the four machine learning algorithms as evaluated by stratified CVs. †) Refers to the AUCs used during the feature selection process. Abbreviations: AE: Adverse events; IK: index keywords; MC: minor drug classes; pct percentile. n(pos): number of positive examples in each drug characteristic dataset.

### Comparative evaluation of cdf with ctf, ctf-icdf, stemming, and drug synonyms

**Figure 4:**
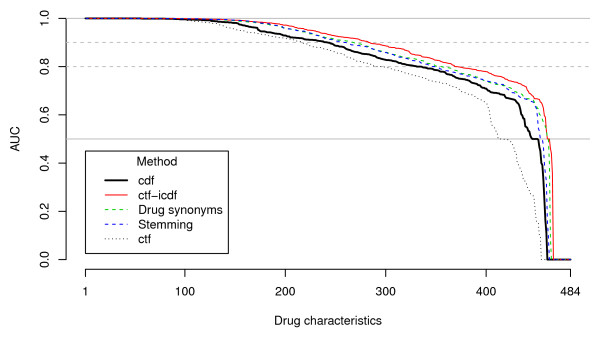
**Comparative performance of cdf with other commonly employed IR methods**. This figure illustrates the predictive performance, assessed by the cross-validation results (in AUC), versus the cumulative number of characteristics (out of 484) for each of the commonly-employed methods. Notes: (1) *ctf: *an average of 1,520 tokens per drug (IQR: 598-3,781 tokens) were retained after elimination. (2) Stemming: the application of stemming algorithm has resulted in the reduction of 20% of tokens (median: 973 tokens per drug, IQR: 455-1,144 tokens). In depth investigation has, however, revealed that stemming did not always group the concept consistently (See additional file 2 for further discussions). (3) Drug synonyms: Compared with MEDLINE searches using only generic names, a mean of 3.4% more results were retrieved when trade names were used (IQR: 0-0.54% more abstracts were retrieved). The dotted lines indicate AUCs of 0.8 and 0.9 respectively. Abbreviations of the method names: *cdf*: conditional document frequency; *ctf*: conditional term frequency; *ctf-icdf*: conditional term frequency-inverse conditional document frequency; Stemming: *cdf *of tokens reduced by Porter's stemming algorithm; Synonyms: *cdf *of tokens generated by retrieving abstracts with both generic and trade names for a given drug.

The classification performance of different IR methods was compared against *cdf *(Figure 4 and Table 5). Both stemming and the incorporation of trade names have resulted in marginal improvements in BICEPP performance (351 and 346 v.s. 330 characteristics predicted with AUC > 0.8). On the other hand, *ctf-icdf *was considerably better when compared with *cdf *(368 characteristics with AUC > 0.8), particularly in the prediction of AMH minor drug class category (73% of characteristics could be predicted with AUC > 0.95). In contrast, using *ctf *for prediction had a significantly poorer result, with only 289 characteristics predicted with AUC > 0.8. The full result set is listed in Additional File 3.

**Table 5:** Comparative evaluation of cdf-based predictions with other commonly used IR methods

Category	Best AUC	Method				
		***cdf***	***ctf***	***ctf-icdf***	**Stemming**	**Synonyms**

AMH major classes	> 0.80	**20 (100)**	**20 (100)**	**20 (100)**	19 (95)	**20 (100)**
	> 0.90	19 (95)	17 (85)	18 (90)	17 (85)	18 (90)
	> 0.95	12 (60)	12 (60)	15 (75)	12 (60)	11 (55)
AMH minor classes	> 0.80	135 (69)	106 (54)*	**157 (80)**	152 (77)	156 (79)
	> 0.90	123 (62)	100 (51)	150 (76)*	145 (74)	151 (77)*
	> 0.95	114 (58)	92 (47)	144 (73)*	142 (72)*	143 (73)*
AMH adverse events	> 0.80	159 (67)	148 (62)	**173 (73)**	153 (64)	155 (65)
	> 0.90	86 (36)	88 (37)	100 (42)	84 (35)	84 (35)
	> 0.95	41 (17)	42 (18)	55 (23)	44 (18)	44 (18)
PKIS perpetrator	> 0.80	7 (47)	6 (40)	7 (47)	**12 (80)**	10 (67)
	> 0.90	5 (33)	5 (33)	4 (27)	3 (20)	4 (27)
	> 0.95	2 (13)	2 (13)	2 (13)	3 (20)	3 (20)
Narrow therapeutic index drugs	> 0.80	9 (64)	9 (64)	**11 (79)**	10 (71)	10 (71)
	> 0.90	8 (57)	7 (50)	10 (71)	9 (64)	10 (71)
	> 0.95	5 (36)	6 (43)	9 (64)	9 (64)	10 (71)

*Overall*	> 0.80	330 (68)	289 (60)*	368 (76)*	346 (71)	351 (73)
	> 0.90	241 (50)	217 (45)	282 (58)*	258 (53)	267 (55)
	> 0.95	174 (36)	154 (32)	225 (46)*	210 (43)	211 (44)

The numbers in this table indicate the number of characteristics (percentage) achieved an AUC above the given thresholds in stratified cross-validation evaluations. For each method, the results from the best of 4 algorithms were compared. The thresholds of AUC can be interpreted as good (> 0.8), very good (> 0.9), and excellent (> 0.95) respectively. The entries labelled (*) indicate a significantly better or worse performance than *cdf *for predicting drug characteristics. Fisher's exact tests were applied as 2 × 2 tables with α = 0.05 adjusted for a family of four comparisons by using the Bonferroni method. The numbers in boldface indicate the best performing method(s) for each characteristic category above the AUC = 0.8 threshold. Abbreviations of the method names: *cdf*: conditional document frequency; *ctf*: conditional term frequency; *ctf-icdf*: conditional term frequency-inverse conditional document frequency; Stemming: *cdf *of tokens reduced by Porter's stemming algorithm; Synonyms: *cdf *of tokens calculated by retrieving abstracts with both generic and trade names for a given drug.

### The predictive performance could be hindered by the heterogeneity within a training set or the lack of knowledge about a drug

We observed two trends in the comparative analyses that were consistent across all IR methods. First, the best AUCs were found to be negatively correlated with the total number of abstracts retrieved from MEDLINE database for drug characteristics with ≥10 positive examples [Item (a), Table 6]; a similar relationship was also observed where the number of positive examples was negatively associated with performance [Items (b), Table 6]. These observations could have resulted from a higher degree of heterogeneity between drugs in a training set with large number of examples (e.g., many drug of different classes can cause the adverse effect of "nausea"). On the other hand, the predictions were generally more accurate when fewer drugs with few abstracts in the MEDLINE database were included in the training set [Items (f) and (h), Table 6]. Because the article count is an indirect indicator of the body of knowledge about a drug, this observation has reinforced the premise that more accurate predictions can be expected if better-studied drug are used in the training of inductive models such as BICEPP.

**Table 6:** The correlations between the training set statistics and BICEPP performance

Training set statistics	Method				
	***cdf***	***ctf***	***ctf-icdf***	**Stemming**	**Synonyms**

**(a) Number of positive examples**	**-.49**	**-.50**	**-.55**	**-.51**	**-.53**
**(b) Sum of article counts**	**-.39**	**-.39**	**-.45**	**-.40**	**-.43**
(c) Maximum article count	-.20	-.19	-.23	-.20	-.26
(d) Mean article count	.09	.10	.06	.10	.00
(e) Median article count	.18	.17	.17	.16	.20
**(f) Minimum article count**	**.32**	**.33**	**.34**	**.34**	**.33**
(g) Variance of articles counts	-.01	-.00	-.03	-.01	-.09
**(h) Skewness of article counts**	**-.33**	**-.35**	**-.36**	**-.35**	**-.40**

The numbers in the table are Spearman's rank correlation coefficients *σ*, calculated by correlating the training set statistics with the best AUC obtained from four machine learning algorithms (NB, IBk, SVM with polynomial and RBF kernels) for each of the 272 of 484 drug characteristics with ≥10 positive examples. For each drug, the corresponding *article count *indicates how many abstracts were retrieved from the MEDLINE database searched by using the drug name. The entries and the category names in boldface indicate *p *< 0.0001 as determined by the test of rank correlation coefficient using the Fieller-Hartley-Pearson method [37]. Abbreviations of the method names: *cdf*: conditional document frequency; *ctf*: conditional term frequency; *ctf-icdf*: conditional term frequency-inverse conditional document frequency; Stemming: *cdf *of tokens reduced by Porter's stemming algorithm; Synonyms: *cdf *of tokens calculated by retrieving abstracts with both generic and trade names for a given drug.

## Discussion

In this study, we have developed a statistical text mining framework for predicting the binary characteristics of biomedical entities using automatically generated features from the MEDLINE database. We have also demonstrated that BICEPP has the potential to predict a wide-range of drug characteristics, including therapeutic classes, indications, adverse effects, and pharmacokinetic drug-drug interactions. While drug properties may be predicted by using other types of data, such as ontology concepts (e.g., unified medical language system, UMLS) [26] or physicochemical properties [27], our approach has an advantage of generalisability because only a common text corpus (i.e., MEDLINE) with a fixed feature selection method is needed to achieve reasonable predictive power. Therefore, BICEPP may be seamlessly adapted to other disciplines to identify unrecognised biomedical relationships without the need to collect additional domain knowledge.

Three points are worthy of note in our approach to drug classification. First, BICEPP makes its predictions only based on a list of examples representing the characteristic of interest. Such "feature-transparent" prediction enables an user to make predictions without the need to know which keywords should be included in literature searches, or which scoring functions should be applied to weigh the importance of the documents retrieved from literature database. Second, the statistical text-mining approach provides a simple alternative to the "deep text-mining" approaches (such as semantic parsing, relationship extraction, and hypothesis generation-based approaches [28-31]), which would require a complex methodology and preprocessing and thus are generally more computationally intensive. With reasonable results shown in our experiments, it is therefore acceptable to use statistical text mining to screen biomedical characteristics on a large scale as demonstrated in our analyses. Third, when using BICEPP to predict the characteristics of a list of drugs, the candidate drugs should be ranked according to the scores produced by the best classifier. Our cross-validation approach can be used to compare the generalisation performance of different classifiers and thus to recommend the best algorithm for the actual predictive task.

We selected clinical pharmacology as the field of study in part because many binary relationships have been systematically collected and extensively replicated in clinical studies. Traditionally, the processes of knowledge synthesis in this field are highly evolved to provide information for clinicians. The clarity of knowledge also provides experimental advantage over other fields such as gene-disease relationships, where more uncertainty exists and replication of data is more limited. In addition, BICEPP may have a role in pharmacovigilance. Our approach could be employed to screen candidate drugs related to adverse events for more in-depth analysis. This would augment existing labour intensive systems such as spontaneous reporting and registries, for instance, the Adverse Event Reporting System (AERS) of the Food and Drug Administration of the United States. In addition, the case for pharmacovigilance application is further strengthened by the example-based approach. Our work have demonstrated that BICEPP may be used to identify obscure textual features (e.g., a keyword denoting a rare genetic variant) that are predictive of a specific aberrant drug behaviour (e.g., an unrecognised adverse event related to a set of pharmacologically unrelated drugs). Such patterns may then be applied to screen candidate drugs with identical patterns in MEDLINE abstracts to institute early warnings and preventative strategies. In practice, BICEPP may be invoked as a component of an *in silico *pharmacovigilance application, such as combining BICEPP with structure-activity relationship analyses [17], to effectively harness the wealth of available biomedical data to achieve better surveillance results. Overall, the applicability of BICEPP's predictions on pharmacovigilance should be further assessed by conducting a prospective surveillance study with rigorous, expert-driven reviews.

Of note, the keywords used to describe a drug characteristic may not be the most discriminative tokens in predicting its association with a drug. This finding is particularly relevant to other text-mining studies in biomedicine, because the co-occurrence of keywords are commonly used (albeit erroneously) to imply associations between biomedical entities. For example, a number of gene-prioritisation tools make empirical assumptions that the co-occurrence of a disease name with a gene symbol implies a potential causal relationship [1,11]. Similarly, keyword co-occurrences have also been applied to identify associations between microbial pathogens and clinical syndromes [32]. Our results demonstrated that this assumption may not always be valid, because the search terms used to describe a biomedical characteristic may be ranked substantially lower compared to other more predictive tokens. A comprehensive review of text features must therefore be conducted if the co-occurrence-based discovery is to be applied in an automated discovery task.

While BICEPP showed promising generalisability in these examples, there are potential limitations and areas to be examined. For instance, BICEPP was evaluated on well-known concepts. It is unknown how BICEPP will perform in a less well known concept to drugs, or in matching characteristics to a new drug. On the other hand, publication bias may skew the distribution of tokens and may represent a strong factor that affects the accuracy of the classification process. For example, a new medication is likely to have fewer publications which may preclude the discovery of discriminative keywords in MEDLINE. Specifically, we have demonstrated that an insufficient corpus could have a detrimental effect on BICEPP's performance. More research is therefore required to investigate how these biases can be effectively addressed to further improve accuracy.

We have evaluated several commonly employed IR methods to study whether BICEPP's predictive performance could be further optimised. Our results have supported the use of *ctf-icdf *for predicting drug properties; the better cross-validation results are consistent with other text-mining tasks employing *tf-idf *[12,18,19]. In particular, *ctf-icdf *tends to outperform *cdf *and other methods when predicting drug characteristics with fewer drug examples such as the category of minor drug classes. Nevertheless, the optimal combinations of these methods require further investigations. For instance, this paper only examined single word tokens for classification, while a more sophisticated tokenisation method with structured analysis of biomedical texts may further improve the discriminative power and should be investigated in future works - such methods may include a combination of n-gram analysis, analysis of ontology, and the incorporation of syntax or grammatical structures as patterns [33,34]. Richer textual patterns may also have better descriptive power in aiding the classification tasks. For example, a comparative study that examined different methods of gene prioritisation showed that methods employing an ontology vocabulary database (including eVOC and MeSH) have superior performance in comparison with other methodologies [35].

The dichotomisation of continuous variables may have reduced the predictive power of BICEPP in some drug characteristics. For instance, the dichotomisation procedure may have resulted in why only 47% of characteristics had AUCs > 0.8 in the category PKIS perpetrators. Previously, we defined perpetrator drugs as drugs capable of altering the plasma concentration of another drug > 2-fold via a CYP450-mediated mechanism. The 2-fold threshold is used by the FDA to classify moderate and strong inhibitors and inducers of drug metabolism [36]. With the imposition of a threshold, drugs that are "borderline significant" (e.g., between 1.5-2 fold change in the target drug concentration) are considered negative examples in the training of BICEPP models. Because the biological mechanisms of DDIs due to these drugs are identical to the perpetrators (i.e., via CYP enzyme inhibition or induction), there may not be tokens sufficiently discriminative to distinguish between the two groups.

## Conclusion

BICEPP is a computational tool that can rapidly identify multiple biomedical entity-characteristic pairs as hypotheses to test in clinical practice or applied research. In this study we have shown that BICEPP predicts known drug properties with reasonable accuracy, and the robustness of BICEPP was demonstrated across a wide range of drug characteristics. The method described in this paper has potential applications in pharmacovigilance and in assisting with the delineation of characteristics in other biomedical disciplines.

## Authors' contributions

FL and SA conceived the project. FL designed BICEPP, collected data, and carried out the experiments. SA contributed to study design and data analysis. MD and TP prepared datasets and interpreted results. GT contributed to the critical discussions. All authors contributed to preparation of the manuscript.

## Supplementary Material

Additional file 1 **Dataset - drug names and drug characteristics studied in this paper**. This file contains the 484 drug characteristic datasets and the list of 857 drug names used to evaluate BICEPP's performance in this paper. Click here for file

Additional file 2 **Supplementary methods and results**. This file describes two analyses on (1) the number of discriminative tokens on BICEPP's predictive performance and (2) discussions on stemming: correlations of CDF between linguistically closely-related tokens. Click here for file

Additional file 3 **Results of cross-validation analysis by algorithms and drug characteristics**. This file contains the full tabular data of cross-validation results summarised in Table 3, Table 5, Figure 2, and Figure 4. Click here for file
