# Supplementary material for: BICEPP: an example-based statistical text mining method for predicting the binary characteristics of drugs
Source: BMC Bioinformatics. 2011 Apr 21;12:112. doi: 10.1186/1471-2105-12-112 (PMC3110144; doi:10.1186/1471-2105-12-112)
Supplement: Additional file 2 — Supplementary methods and results. This file describes two analyses on (1) the number of discriminative tokens on BICEPP's predictive performance and (2) discussions on stemming: correlations of CDF between linguistically closely-related tokens. [file 1471-2105-12-112-S2.PDF]

BInary Characteristic Extractor and Property Predictor (BICEPP): an example-based statistical text mining method for predicting the binary characteristics of drugs

Additional File 2: Supplementary methods and results

Authors: Frank Po-Yen Lin[1*], Stephen Anthony[1], Thomas M. Polasek[2], Guy Tsafnat[2], Matthew P. Doogue[2,3]

1. Centre for Health Informatics, The University of New South Wales, Sydney, Australia
2. Department of Clinical Pharmacology, Flinders University, Adelaide, Australia
3. Flinders Medical Centre, Adelaide, Australia

The effect of number of discriminative tokens on predictive performance

Motivation and Methods

The effect of number of discriminative tokens on BICEPP performance is unknown. To select the number of predictive tokens for comparative evaluations, we applied forward feature selection procedure to evaluate 15 drug characteristics. Each of the 15 drug characteristics had $\geq$10 positive examples and the best AUC of CDF $>$0.9. We iteratively added between 1 to 100 most discriminative tokens from the top of the token rank. The conditional document frequency (CDF) of the top-n tokens were used to predict drug characteristics. Classification performance was estimated by calculating the medians from 5 $\times$ 10-fold cross-validations. Feature selection was performed only on the training folds to avoid overestimation of classification accuracy.

Results

From the 15 drug characteristics evaluated, a general trend was observed such that the performance improved when n is incrementally increased to 20 (Figure 1). It can also be observed that the best classification performance could be achieved when top 20–50 tokens were used for classification. In some cases, however, a reduction of classifier performances was observed when lower-ranked tokens were also included for classification (n =50–100). This effect can be seen in cases of “bruising“ (case 4), “antidote“ (case 10), “CYP2D6 inhibitors“ (case 12), and “CYP3A4 inhibitors” (case 13). Variability between algorithms were also observed.

Discussion

The best n for optimal prediction requires assessments on a case-by-case basis. Based on these findings, we elected to use a fixed threshold to permit consistent comparison across drug characteristics and individual algorithms. It is expected that a threshold of 20 top-ranked tokens should yield a reasonable classification power for conducting comparative evaluations.

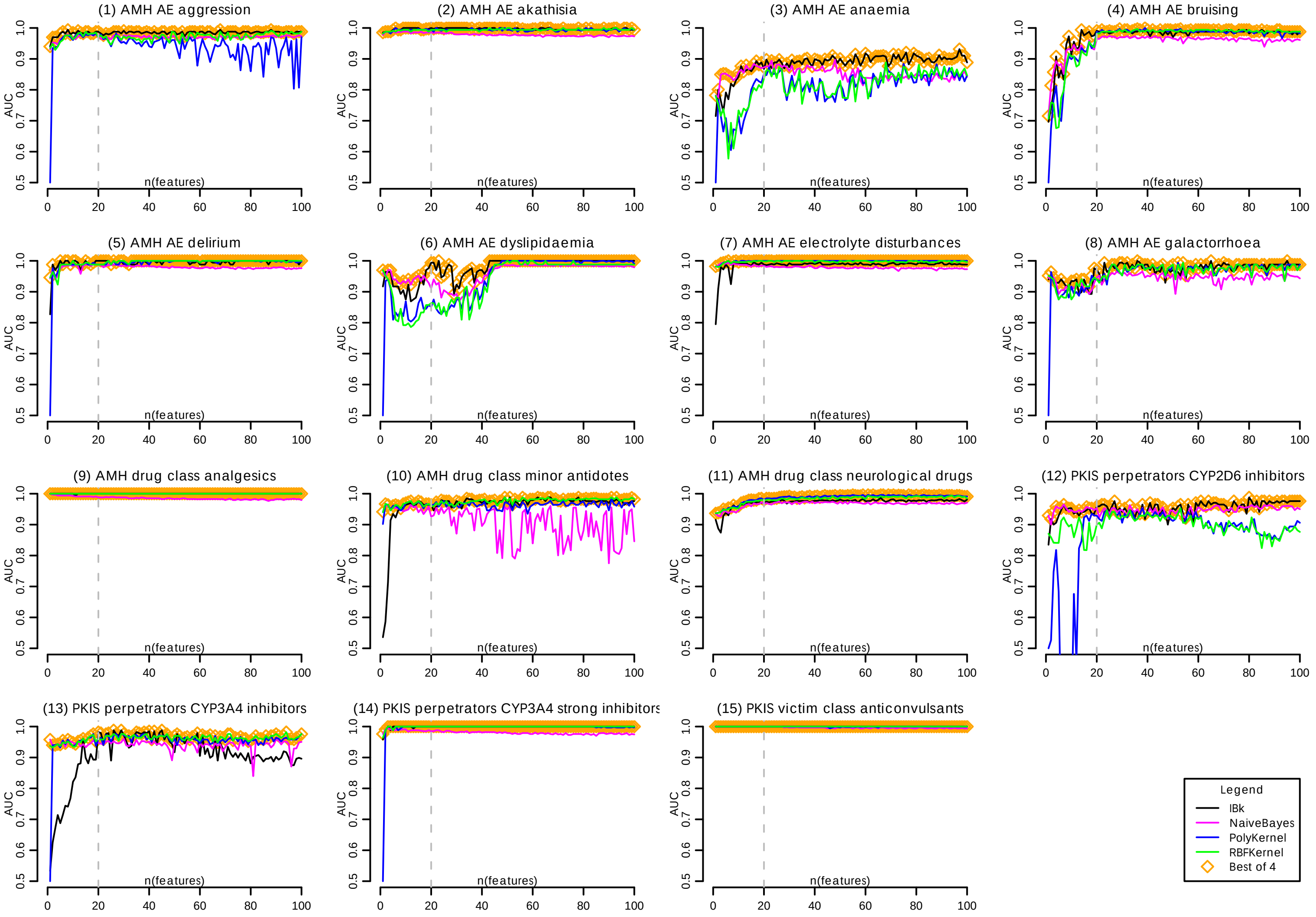

Figure 1: **Classification performance v.s. number of discriminative tokens** (n) **by classifiers.** The grey dashed lines indicate n=20.

Discussion on stemming

Motivation and method

Stemming is an important technique in information retrieval (IR) systems to improve recall of document retrieval. A stemmer aggregates closely-related words derived from the same linguistic origin. We found that, however, stemming failed to group concepts consistently for our tasks. Here we analysed the correlations between top-200 tokens of characteristic "anti-infectives" (AMH drug class). The drug characteristic has 135 positive examples and a best AUC of 0.985 (see Additional File 3). We calculated Pearson's correlations between CDFs of tokens that are potential candidates for stemming.

Results

Pairwise comparisons revealed that the majority of token pairs have $r^2 > 0.8$. For example, highly-correlated token pairs, such as "antibiotic" vs "antibiotics" ($r^2 = 0.98$), are meaningful candidates to apply stemming methods. In contrast, a significant proportion of stemmable token-pairs have poor correlations (Table 1), including "disseminated" vs. "dissemination" ($r^2 = 0.47$), "infecting" vs. "infection", "infectious", and "infected" (r^2 =0.3–0.5), "south" vs. "southern" ($r^2 = 0.57$), and "confer" vs. "conferring" ($r^2 = 0.52$).

The derived tokens also showed high variabilities in their discriminative power (Table 2). For example, for the words stemmed from "resist", the word "resistant" had a good AUC of 0.95, whereas the word "resistances" had only a poor AUC of 0.68.

Discussion

In contrast to the goal of conventional IR tasks, the use of stemming in BICEPP may reduce the specificities of otherwise predictive tokens. This may be due to the fact that the concepts represented by individual tokens are used in very different biomedical contexts.

Table 1: Correlation of CDFs between linguistically closely-related tokens in AMH drug class of anti-infectives

Token 1	Token 2	r^2
mic	mics	0.98
antibiotics	antibiotic	0.98
macrolide	macrolides	0.97
isolates	isolate	0.95
susceptibility	susceptible	0.95
bacterial	bacteria	0.94
beta-lactamase	beta-lactam	0.93
staphylococcus	staphylococci	0.93
susceptibility	susceptibilities	0.93
antimicrobial	antimicrobials	0.92
abscess	abscesses	0.91
mics	mic90	0.90
mic	mic90	0.90
empirical	empiric	0.89
susceptible	susceptibilities	0.89
regimens	regimen	0.89
eradication	eradicated	0.89
aminoglycosides	aminoglycoside	0.89
virus	viral	0.88
pathogens	pathogen	0.83
organisms	organism	0.83
eradication	eradicate	0.82
infected	infection	0.82
eradicate	eradicated	0.82
microbiological	microbiology	0.82
inoculation	inoculated	0.81
tetracycline	tetracyclines	0.79
streptococci	streptococcus	0.78
infection	infections	0.77
prophylaxis	prophylactic	0.74
cure	cured	0.73
infection	infectious	0.67
dilution	dilutions	0.65
rifampicin	rifampin	0.65
infections	infecting	0.62
pneumonia	pneumoniae	0.61
infections	infectious	0.61
hospitalized	hospitals	0.60
south	southern	0.57
infected	infections	0.54
conferring	confer	0.52
emergence	emerged	0.48
infected	infectious	0.48
disseminated	dissemination	0.47
infection	infecting	0.45
infectious	infecting	0.36
infected	infecting	0.32
inoculated	inoculum	0.30

Table 2: The AUCs of closely-related words in predicting whether a drug belongs to AMH drug class of anti-infectives

Root	Derived words
Resist	resistant (0.95), resistances (0.68), resist (0.51), resisted (0.50)
Infect	infected (0.97), infection (0.96), infections (0.95), infectious (0.89), infecting (0.73), infective (0.70), infectivity (0.61), infect (0.59), infects (0.53)
Empiric	empirical (0.76), empiric (0.72), empirically (0.65)
Eradicate	eradication (0.82), eradicate (0.74), eradicated (0.73), eradicating (0.60), eradicates (0.51)
