# Supplementary material for: BICEPP: an example-based statistical text mining method for predicting the binary characteristics of drugs
Source: BMC Bioinformatics. 2011 Apr 21;12:112. doi: 10.1186/1471-2105-12-112 (PMC3110144; doi:10.1186/1471-2105-12-112)
Supplement: Additional file 3 — Results of cross-validation analysis by algorithms and drug characteristics. This file contains the full tabular data of cross-validation results summarised in Table 3, Table 5, Figure 2, and Figure 4. [file 1471-2105-12-112-S3.PDF]

BInary Characteristic Extractor and Property Predictor (BICEPP): an example-based statistical text mining method for predicting the binary characteristics of drugs

Additional File 3: Stratified cross-validation results by algorithms and drug characteristics

Authors: Frank Po-Yen Lin[1*], Stephen Anthony[1], Thomas M. Polasek[2], Guy Tsafnat[2], Matthew P. Doogue[2,3]

1. Centre for Health Informatics, The University of New South Wales, Sydney, Australia
2. Department of Clinical Pharmacology, Flinders University, Adelaide, Australia
3. Flinders Medical Centre, Adelaide, Australia

Abbreviations

AE: adverse events
AMH: Australian Medicines Handbook
AUC: area(s) under the receiver operating characteristic curve
cdf: conditional document frequency
ctf: conditional term frequency
ctf-icdf: conditional term frequency-inverse conditional document frequency
CYP: cytochromes P450
Drug synonyms: *cdf* of tokens calculated by retrieving abstracts with both generic and trade names for a given drug
IBk: *k*-nearest neighbour algorithm
NB: Naïve Bayes
PKIS: PharmacoKinetic Interaction Screening database
Stemming: *cdf* of tokens reduced by Porter's stemming algorithm
SVM: support vector machine
SVL: linear SVM
SVR: support vector machine with radial basis function kernel

Table A3. Cross-validation results by algorithms and drug characteristics

		AUC (method / algorithm)																			
		cdf				*ctf*				*cdf-icdf*				Stemming				Drug synonyms			
Characteristic name	n	NB	IBk	SVL	SVR	NB	IBk	SVL	SVR	NB	IBk	SVL	SVR	NB	IBk	SVL	SVR	NB	IBk	SVL	SVR
AMH AE abdominal cramps	12	.614	.670	.501	.522	.631	.561	.586	.588	.651	.384	.496	.470	.519	.599	.590	.578	.704	.740	.685	.671
AMH AE abdominal pain	142	.624	.650	.631	.640	.634	.649	.607	.608	.675	.740	.620	.634	.647	.702	.618	.636	.658	.683	.649	.647
AMH AE abnormal liver enzymes	25	.827	.823	.759	.747	.809	.821	.723	.691	.873	.921	.846	.842	.809	.776	.762	.751	.825	.779	.777	.762
AMH AE acne	33	.867	.858	.849	.883	.854	.831	.842	.879	.841	.888	.820	.851	.840	.843	.849	.851	.851	.861	.851	.846
AMH AE aggression	16	.970	.981	.901	.968	.901	.977	.878	.950	.964	.981	.969	.973	.955	.972	.875	.936	.955	.974	.944	.946
AMH AE agitation	70	.863	.862	.797	.860	.857	.840	.833	.865	.871	.861	.827	.873	.865	.820	.823	.855	.865	.841	.807	.854
AMH AE agranulocytosis	46	.653	.689	.641	.666	.682	.724	.714	.713	.740	.741	.721	.731	.679	.740	.721	.735	.704	.743	.760	.756
AMH AE akathisia	20	.981	.992	.987	.989	.966	.989	.975	.982	.978	.988	.979	.983	.947	.976	.969	.973	.979	.990	.976	.987
AMH AE allergic contact dermatitis	17	.807	.796	.804	.785	.804	.795	.811	.796	.780	.864	.799	.803	.798	.825	.809	.781	.824	.824	.799	.791
AMH AE allergic skin reactions	11	.775	.801	.793	.806	.769	.763	.792	.770	.799	.784	.843	.846	.795	.773	.906	.911	.776	.781	.901	.886
AMH AE alopecia	95	.805	.790	.788	.807	.801	.816	.801	.807	.800	.817	.765	.762	.792	.801	.768	.774	.742	.752	.691	.707
AMH AE amenorrhoea	26	.905	.887	.881	.898	.906	.888	.850	.915	.862	.932	.887	.883	.853	.868	.822	.855	.896	.926	.877	.899
AMH AE anaemia	40	.847	.848	.776	.770	.842	.858	.776	.755	.879	.873	.861	.858	.862	.871	.800	.807	.830	.849	.659	.669
AMH AE anaphylaxis or anaphylatoid reactions	205	.719	.735	.745	.741	.733	.768	.760	.752	.760	.789	.751	.767	.742	.767	.767	.765	.733	.728	.738	.751
AMH AE angina	17	.783	.730	.844	.858	.779	.776	.745	.775	.850	.876	.871	.875	.859	.768	.839	.843	.809	.726	.849	.859
AMH AE angioedema	106	.727	.765	.740	.750	.739	.767	.749	.739	.758	.762	.750	.754	.765	.774	.794	.786	.760	.772	.750	.765
AMH AE anorexia	83	.687	.722	.603	.515	.699	.730	.629	.611	.760	.755	.700	.685	.680	.722	.577	.513	.694	.726	.556	.543
AMH AE anxiety	71	.889	.894	.910	.911	.890	.909	.911	.911	.915	.917	.848	.903	.881	.863	.876	.895	.877	.859	.901	.905
AMH AE aplastic anaemia	30	.835	.834	.786	.820	.810	.847	.773	.843	.815	.839	.784	.780	.798	.814	.698	.692	.785	.840	.731	.739
AMH AE arrhythmias	75	.804	.751	.740	.752	.799	.797	.709	.728	.790	.826	.684	.687	.784	.780	.672	.677	.800	.717	.661	.660
AMH AE arthralgia	70	.627	.685	.608	.611	.598	.681	.541	.562	.686	.735	.642	.635	.621	.661	.566	.557	.652	.673	.618	.604
AMH AE arthritis	18	.816	.826	.663	.645	.815	.839	.745	.716	.831	.905	.745	.728	.838	.846	.669	.649	.842	.827	.788	.807
AMH AE aseptic meningitis	18	.849	.937	.843	.899	.844	.948	.869	.866	.928	.944	.934	.927	.834	.940	.863	.858	.836	.921	.838	.852
AMH AE ataxia	36	.806	.784	.756	.799	.814	.788	.807	.814	.824	.840	.781	.825	.812	.772	.786	.802	.808	.799	.807	.815
AMH AE atrioventricular block	9	.110	.110	.111	.111	0	0	0	0	.820	.833	.839	.839	0	0	0	0	0	0	0	0

(Continue on next page)

Table A3. Cross-validation results by algorithms and drug characteristics (cont'd)

		AUC (method / algorithm)																			
		cdf				*ctf*				*cdf-icdf*				Stemming				Drug synonyms			
Characteristic name	n	NB	IBk	SVL	SVR	NB	IBk	SVL	SVR	NB	IBk	SVL	SVR	NB	IBk	SVL	SVR	NB	IBk	SVL	SVR
AMH AE back pain	18	.716	.769	.763	.765	.749	.816	.779	.766	.770	.816	.690	.702	.743	.703	.776	.785	.803	.758	.760	.771
AMH AE bilirubin	25	.775	.883	.784	.788	.799	.863	.682	.738	.853	.876	.870	.871	.777	.765	.667	.739	.748	.747	.702	.738
AMH AE blood disorders	14	.748	.743	.757	.761	.767	.763	.771	.773	.948	.951	.986	.983	.881	.873	.906	.911	.081	.093	.091	.096
AMH AE blood dyscrasias	70	.806	.864	.815	.814	.861	.906	.866	.865	.867	.887	.878	.870	.803	.828	.825	.823	.816	.834	.834	.838
AMH AE blurred vision	86	.731	.750	.648	.636	.749	.728	.601	.615	.785	.789	.712	.723	.763	.780	.649	.672	.784	.762	.676	.698
AMH AE bone pain	14	.910	.911	.908	.906	.934	.907	.925	.922	.929	.938	.948	.947	.958	.927	.914	.919	.968	.905	.905	.905
AMH AE bradycardia	49	.838	.772	.788	.793	.853	.855	.844	.840	.847	.838	.809	.801	.837	.776	.806	.805	.833	.803	.831	.832
AMH AE breast enlargement	19	.814	.776	.841	.852	.788	.744	.836	.829	.869	.875	.821	.826	.824	.821	.848	.852	.851	.887	.874	.860
AMH AE bronchospasm	89	.769	.779	.706	.703	.762	.786	.717	.729	.792	.781	.720	.727	.696	.689	.654	.676	.751	.759	.694	.690
AMH AE bruising	18	.892	.918	.937	.934	.827	.861	.867	.843	.864	.879	.868	.860	.882	.891	.877	.874	.848	.889	.871	.868
AMH AE burning	25	.886	.875	.805	.801	.870	.874	.841	.780	.873	.876	.827	.827	.870	.864	.749	.747	.824	.708	.750	.745
AMH AE cardiac arrest	13	.843	.847	.838	.825	.897	.887	.881	.881	.850	.864	.880	.859	.832	.802	.794	.842	.831	.838	.795	.826
AMH AE chest pain	40	.734	.768	.757	.756	.745	.734	.785	.777	.780	.751	.750	.745	.746	.764	.804	.804	.775	.774	.845	.843
AMH AE chills	18	.800	.899	.695	.697	.807	.929	.703	.666	.866	.857	.782	.810	.792	.824	.561	.738	.786	.884	.578	.746
AMH AE chloasma	12	.918	.865	.917	.914	.920	.862	.833	.834	.852	.968	.746	.795	.860	.927	.814	.826	.850	.942	.815	.823
AMH AE cholestatic jaundice	14	.843	.881	.764	.743	.853	.847	.885	.881	.874	.914	.847	.841	.846	.888	.874	.854	.820	.871	.804	.803
AMH AE clostridium difficile associated disease	47	.981	.991	.994	.993	.985	.993	.994	.993	.987	.996	.996	.995	.987	.991	.995	.992	.986	.993	.995	.993
AMH AE confusion	114	.770	.761	.791	.790	.762	.754	.789	.789	.781	.788	.755	.760	.757	.764	.786	.772	.762	.777	.769	.775
AMH AE conjunctivitis	12	.807	.873	.585	.561	.833	.881	.580	.624	.868	.772	.817	.834	.827	.839	.430	.431	.761	.753	.487	.439
AMH AE constipation	154	.672	.712	.658	.656	.663	.674	.639	.654	.706	.793	.721	.719	.652	.691	.622	.629	.662	.693	.611	.646
AMH AE contact dermatitis	18	.920	.918	.927	.929	.912	.926	.940	.934	.921	.930	.932	.926	.931	.917	.944	.944	.869	.883	.847	.863
AMH AE cough	59	.703	.802	.608	.728	.674	.756	.643	.714	.719	.723	.737	.738	.668	.707	.662	.706	.691	.754	.680	.727
AMH AE cystitis	13	.993	.999	1	1	.991	.999	.999	1	.990	.998	.998	.999	.993	.998	.999	.999	.993	.999	1	.999
AMH AE delayed wound healing	10	.985	.995	.992	.996	.984	.992	.991	.995	.989	.998	.897	.995	.946	.980	.967	.985	.982	.997	.972	.997
AMH AE delirium	14	.855	.947	.859	.844	.856	.950	.913	.932	.918	.942	.967	.949	.855	.957	.912	.943	.904	.949	.868	.869

(Continue on next page)

Table A3. Cross-validation results by algorithms and drug characteristics (cont'd)

		AUC (method / algorithm)																			
		cdf				*ctf*				*cdf-icdf*				Stemming				Drug synonyms			
Characteristic name	n	NB	IBk	SVL	SVR	NB	IBk	SVL	SVR	NB	IBk	SVL	SVR	NB	IBk	SVL	SVR	NB	IBk	SVL	SVR
AMH AE depression	67	.673	.733	.634	.625	.697	.741	.636	.640	.714	.716	.645	.647	.689	.757	.676	.689	.693	.740	.664	.664
AMH AE dermatitis	13	.587	.534	.747	.713	.620	.647	.657	.629	.604	.684	.545	.540	.551	.498	.505	.528	.525	.438	.526	.533
AMH AE diarrhoea	308	.699	.743	.727	.722	.687	.735	.740	.732	.742	.779	.779	.754	.681	.734	.715	.705	.693	.750	.737	.717
AMH AE disorientation	15	.729	.751	.779	.763	.678	.674	.710	.698	.918	.904	.880	.885	.583	.626	.626	.618	0	0	0	0
AMH AE dizziness	286	.690	.696	.696	.693	.675	.685	.687	.688	.750	.771	.751	.746	.701	.710	.712	.706	.724	.722	.734	.728
AMH AE drowsiness	92	.815	.804	.763	.794	.803	.831	.777	.811	.825	.824	.809	.826	.811	.799	.789	.801	.798	.796	.759	.772
AMH AE drug fever	13	.919	.933	.875	.884	.921	.934	.880	.964	.924	.920	.863	.864	.919	.921	.865	.872	.893	.906	.886	.873
AMH AE dry eyes	10	.435	.623	.647	.613	.572	.754	.680	.685	.629	.753	.688	.708	.484	.602	.582	.579	.508	.640	.657	.637
AMH AE dry mouth	115	.835	.809	.811	.831	.839	.804	.807	.836	.861	.890	.840	.854	.841	.839	.835	.836	.858	.839	.854	.856
AMH AE dry skin	22	.658	.716	.677	.701	.704	.730	.716	.696	.779	.783	.712	.683	.657	.732	.661	.650	.689	.761	.684	.678
AMH AE dyskinesia	14	.852	.811	.688	.680	.772	.871	.703	.703	.910	.978	.819	.804	.850	.740	.790	.731	.785	.790	.737	.645
AMH AE dyslipidaemia	13	.900	.891	.775	.767	.911	.934	.854	.880	.923	.958	.927	.933	.804	.906	.825	.829	.973	.963	.949	.950
AMH AE dyspepsia	76	.717	.725	.731	.703	.693	.717	.704	.685	.761	.805	.753	.755	.673	.718	.678	.633	.683	.743	.700	.693
AMH AE dyspnoea	63	.655	.663	.624	.657	.684	.643	.661	.674	.725	.781	.741	.738	.736	.775	.721	.748	.656	.666	.624	.653
AMH AE dystonia	12	.922	.969	.955	.975	.886	.914	.889	.925	.957	.974	.975	.983	.939	.977	.909	.974	.935	.972	.905	.980
AMH AE electrolyte disturbances	25	.922	.926	.935	.938	.913	.902	.942	.936	.925	.934	.931	.951	.912	.883	.944	.954	.917	.844	.947	.940
AMH AE encephalopathy	11	.733	.787	.582	.702	.580	.634	.655	.574	.724	.767	.593	.622	.685	.695	.508	.599	.775	.783	.577	.728
AMH AE eosinophilia	37	.802	.811	.732	.693	.758	.800	.631	.637	.827	.808	.797	.800	.813	.808	.614	.592	.784	.804	.704	.718
AMH AE epse	24	.946	.993	.989	.988	.945	.990	.985	.986	.970	.992	.961	.985	.943	.993	.992	.987	.956	.995	.994	.990
AMH AE erythema	57	.808	.886	.739	.698	.771	.822	.772	.770	.822	.848	.775	.760	.764	.830	.757	.757	.824	.839	.793	.798
AMH AE euphoria	18	.941	.962	.871	.935	.950	.955	.905	.951	.953	.950	.955	.959	.944	.954	.916	.925	.949	.958	.884	.920
AMH AE exfoliative dermatitis	33	.756	.797	.677	.676	.732	.757	.526	.523	.784	.741	.748	.748	.713	.701	.661	.657	.726	.651	.684	.692
AMH AE facial flushing	102	.747	.749	.749	.747	.749	.744	.753	.749	.754	.784	.686	.694	.810	.805	.770	.771	.736	.749	.729	.729
AMH AE fatigue	118	.735	.742	.748	.728	.713	.734	.743	.727	.736	.762	.684	.683	.714	.730	.722	.707	.753	.747	.746	.739
AMH AE fever	115	.730	.717	.624	.625	.722	.708	.600	.597	.736	.735	.577	.609	.710	.674	.565	.614	.691	.665	.470	.548

(Continue on next page)

Table A3. Cross-validation results by algorithms and drug characteristics (cont'd)

		AUC (method / algorithm)																			
		cdf				*ctf*				*cdf-icdf*				Stemming				Drug synonyms			
Characteristic name	n	NB	IBk	SVL	SVR	NB	IBk	SVL	SVR	NB	IBk	SVL	SVR	NB	IBk	SVL	SVR	NB	IBk	SVL	SVR
AMH AE flatulence	26	.789	.827	.658	.686	.629	.770	.626	.643	.770	.772	.669	.694	.771	.761	.738	.737	.658	.825	.598	.623
AMH AE flu like symptoms	18	.912	.890	.874	.872	.928	.900	.903	.897	.896	.921	.848	.846	.891	.898	.882	.889	.922	.888	.895	.896
AMH AE fluid retention	30	.910	.892	.856	.830	.906	.879	.850	.882	.872	.885	.833	.845	.891	.901	.869	.872	.872	.913	.813	.828
AMH AE fractures	12	.962	.968	.969	.970	.955	.962	.942	.943	.961	.979	.976	.977	.922	.916	.943	.937	.941	.979	.959	.956
AMH AE galactorrhoea	13	.852	.936	.832	.867	.832	.928	.881	.860	.897	.938	.852	.836	.878	.917	.920	.912	.911	.928	.842	.841
AMH AE gastritis	23	.897	.921	.904	.892	.904	.914	.879	.866	.939	.946	.948	.948	.920	.881	.929	.912	.915	.899	.904	.886
AMH AE gi haemorrhage	17	.644	.670	.458	.496	.661	.728	.610	.557	.723	.588	.666	.653	.555	.677	.537	.526	.606	.727	.652	.637
AMH AE gi ulceration or haemorrhage	14	.993	.998	.999	.999	.991	.998	.999	.999	.991	.998	.998	.998	.993	.999	.998	.997	.992	.998	.999	.998
AMH AE gingival hyperplasia	9	0	0	0	0	0	0	0	0	.801	.819	.827	.826	0	0	0	0	0	0	0	0
AMH AE glaucoma	11	.988	1	.998	.997	.990	.997	.997	.997	.984	.999	.999	.998	.949	.988	.975	.981	.983	.996	.958	.996
AMH AE glossitis	11	.860	.842	.849	.849	.942	.950	.980	.966	.856	.879	.888	.896	.866	.878	.884	.882	.830	.817	.809	.838
AMH AE gynaecomastia	37	.804	.825	.806	.792	.810	.856	.760	.770	.831	.837	.802	.807	.802	.833	.849	.845	.818	.870	.844	.848
AMH AE haemolytic anaemia	50	.797	.832	.741	.731	.758	.800	.674	.642	.818	.816	.791	.789	.767	.757	.736	.726	.770	.750	.743	.730
AMH AE haemorrhage	50	.889	.891	.891	.895	.880	.865	.879	.871	.872	.865	.871	.867	.884	.868	.852	.856	.841	.839	.844	.830
AMH AE hallucinations	56	.689	.693	.631	.641	.723	.730	.664	.693	.735	.759	.618	.614	.650	.688	.602	.633	.665	.726	.561	.651
AMH AE headache	405	.617	.623	.639	.632	.612	.647	.651	.635	.662	.720	.664	.650	.614	.625	.627	.619	.625	.639	.656	.627
AMH AE heart failure	46	.741	.766	.712	.725	.733	.760	.740	.745	.782	.786	.791	.790	.726	.728	.735	.746	.743	.755	.728	.746
AMH AE hepatitis	138	.745	.785	.768	.771	.731	.774	.759	.759	.777	.797	.794	.796	.727	.768	.630	.720	.739	.767	.743	.758
AMH AE hepatotoxicity	53	.602	.619	.585	.624	.601	.704	.494	.638	.720	.664	.738	.726	.619	.640	.551	.616	.635	.591	.575	.588
AMH AE hirsutism	20	.918	.882	.851	.907	.919	.879	.794	.831	.869	.943	.803	.804	.870	.900	.853	.847	.879	.916	.850	.841
AMH AE hypercholesterolaemia	15	.872	.961	.906	.934	.886	.942	.901	.913	.929	.937	.928	.939	.924	.934	.944	.939	.945	.948	.938	.939
AMH AE hyperglycaemia	48	.635	.778	.721	.733	.663	.765	.687	.726	.796	.819	.821	.824	.650	.723	.676	.708	.678	.760	.678	.687
AMH AE hyperkalaemia	42	.865	.928	.856	.883	.830	.904	.840	.851	.870	.895	.881	.879	.857	.931	.835	.855	.884	.913	.874	.884
AMH AE hyperprolactinaemia	22	.976	.990	.994	.993	.977	.991	.993	.993	.977	.991	.994	.992	.977	.990	.984	.992	.977	.990	.981	.989
AMH AE hypersalivation	14	.975	.983	.967	.980	.966	.978	.854	.977	.975	.983	.984	.983	.974	.988	.943	.981	.973	.979	.975	.975

(Continue on next page)

Table A3. Cross-validation results by algorithms and drug characteristics (cont'd)

		AUC (method / algorithm)																			
		cdf				*ctf*				*cdf-icdf*				Stemming				Drug synonyms			
Characteristic name	n	NB	IBk	SVL	SVR	NB	IBk	SVL	SVR	NB	IBk	SVL	SVR	NB	IBk	SVL	SVR	NB	IBk	SVL	SVR
AMH AE hypersensitivity	303	.656	.685	.654	.649	.625	.670	.633	.627	.639	.702	.656	.656	.634	.669	.641	.633	.626	.689	.640	.618
AMH AE hypertension	105	.707	.735	.654	.676	.705	.731	.646	.672	.720	.783	.686	.687	.712	.729	.660	.683	.711	.738	.696	.708
AMH AE hypertriglyceridaemia	16	.902	.929	.833	.903	.925	.948	.898	.927	.906	.929	.882	.906	.917	.930	.881	.913	.908	.916	.857	.911
AMH AE hyperuricaemia	14	.764	.796	.753	.777	.685	.772	.765	.729	.741	.821	.729	.737	.693	.703	.742	.703	.785	.835	.874	.849
AMH AE hypocalcaemia	15	.782	.815	.814	.786	.717	.764	.781	.783	.921	.921	.926	.934	.853	.917	.888	.916	.782	.887	.738	.844
AMH AE hypoglycaemia	14	.834	.919	.846	.910	.831	.895	.738	.922	.851	.886	.718	.830	.821	.762	.781	.872	.835	.867	.737	.854
AMH AE hypokalaemia	38	.757	.779	.753	.762	.729	.742	.740	.733	.780	.812	.789	.791	.652	.731	.654	.656	.713	.777	.698	.690
AMH AE hypomagnesaemia	17	.791	.866	.869	.845	.822	.829	.919	.915	.926	.937	.951	.944	.851	.873	.848	.855	.854	.878	.805	.844
AMH AE hyponatraemia	29	.873	.942	.856	.857	.830	.923	.866	.837	.878	.893	.885	.885	.853	.908	.909	.889	.810	.903	.881	.877
AMH AE hypophosphataemia	15	.817	.832	.911	.907	.831	.884	.902	.902	.883	.893	.816	.856	.898	.941	.939	.929	.895	.938	.914	.935
AMH AE hypotension	138	.715	.727	.742	.738	.691	.717	.708	.705	.721	.755	.662	.664	.689	.706	.728	.725	.690	.700	.707	.703
AMH AE impotence	41	.776	.837	.796	.803	.767	.875	.843	.852	.871	.857	.892	.895	.764	.838	.803	.814	.744	.821	.770	.751
AMH AE increased appetite	12	.806	.892	.748	.744	.823	.798	.714	.720	.790	.848	.821	.820	.803	.794	.697	.732	.817	.756	.822	.827
AMH AE increased creatinine concentration	17	.795	.823	.787	.787	.780	.797	.779	.785	.897	.923	.916	.907	.775	.833	.800	.811	.782	.840	.784	.802
AMH AE increased liver enzymes	181	.721	.745	.703	.722	.710	.738	.717	.734	.755	.781	.740	.743	.711	.723	.697	.710	.713	.728	.694	.701
AMH AE infections	25	.944	.946	.933	.943	.925	.900	.899	.901	.966	.924	.962	.966	.946	.946	.929	.941	.956	.949	.922	.938
AMH AE infertility	12	.890	.893	.879	.899	.988	.996	.989	.984	.990	.994	1	1	.989	.994	.998	.973	.989	.992	.998	.979
AMH AE inflammation at injection site	23	.969	.984	.953	.967	.971	.986	.952	.983	.958	.984	.951	.952	.957	.984	.951	.950	.963	.985	.944	.950
AMH AE injection site reactions	36	.777	.697	.701	.708	.790	.719	.751	.724	.793	.793	.722	.730	.763	.730	.647	.649	.786	.759	.680	.673
AMH AE insomnia	116	.774	.786	.739	.749	.761	.753	.716	.732	.767	.834	.745	.736	.763	.751	.747	.751	.760	.761	.741	.746
AMH AE interstitial nephritis	58	.892	.920	.837	.861	.891	.927	.810	.862	.892	.917	.919	.915	.864	.907	.847	.866	.885	.918	.930	.927
AMH AE irritability	15	.790	.753	.630	.641	.696	.657	.624	.634	.848	.818	.806	.804	.773	.724	.674	.699	.695	.561	.723	.739
AMH AE irritation	12	.910	.903	.804	.805	.858	.920	.857	.848	.916	.915	.750	.762	.849	.916	.786	.795	.828	.801	.728	.766
AMH AE itching	162	.671	.700	.683	.669	.652	.674	.692	.673	.667	.698	.641	.642	.669	.675	.684	.682	.687	.692	.665	.664
AMH AE jaundice	48	.691	.685	.674	.668	.656	.696	.653	.654	.665	.735	.620	.641	.680	.697	.616	.636	.587	.627	.560	.559

(Continue on next page)

Table A3. Cross-validation results by algorithms and drug characteristics (cont'd)

		AUC (method / algorithm)																			
		cdf				*ctf*				*cdf-icdf*				Stemming				Drug synonyms			
Characteristic name	n	NB	IBk	SVL	SVR	NB	IBk	SVL	SVR	NB	IBk	SVL	SVR	NB	IBk	SVL	SVR	NB	IBk	SVL	SVR
AMH AE lactic acidosis	14	.702	.792	.725	.700	.754	.727	.857	.853	.872	.951	.908	.915	.860	.906	.902	.904	.794	.814	.885	.883
AMH AE lethargy	13	.714	.703	.746	.749	.761	.725	.742	.712	.750	.788	.687	.690	.663	.656	.741	.744	.860	.884	.723	.676
AMH AE leucocytosis	12	.866	.858	.870	.860	.867	.857	.790	.858	.913	.918	.887	.916	.807	.820	.832	.771	.763	.766	.768	.768
AMH AE leucopenia	73	.684	.694	.667	.709	.717	.733	.759	.766	.729	.759	.654	.653	.650	.687	.604	.649	.659	.732	.648	.687
AMH AE libido changes	51	.868	.904	.881	.869	.858	.894	.889	.880	.893	.907	.907	.900	.858	.918	.881	.871	.875	.906	.893	.886
AMH AE liver failure	31	.832	.796	.850	.849	.780	.802	.807	.816	.842	.824	.851	.850	.784	.760	.836	.833	.771	.785	.817	.824
AMH AE maculopapular rash	13	.920	.941	.943	.945	.931	.953	.951	.950	.960	.948	.899	.907	.929	.949	.944	.946	.923	.948	.867	.942
AMH AE malaise	23	.864	.907	.553	.617	.872	.923	.781	.762	.905	.915	.817	.781	.893	.910	.727	.750	.841	.904	.640	.622
AMH AE mania	12	.951	.971	.891	.929	.951	.969	.852	.950	.966	.976	.959	.981	.961	.981	.920	.980	.950	.955	.966	.958
AMH AE migraine	14	.906	.906	.794	.847	.861	.911	.845	.863	.922	.926	.894	.884	.895	.908	.768	.839	.818	.867	.851	.847
AMH AE miosis	13	.947	.980	.967	.967	.958	.983	.965	.969	.958	.987	.975	.977	.939	.984	.957	.965	.923	.973	.953	.956
AMH AE mood changes	31	.844	.804	.735	.748	.861	.834	.809	.799	.817	.851	.823	.813	.825	.853	.766	.776	.828	.855	.807	.800
AMH AE muscle cramps	49	.895	.866	.913	.895	.881	.847	.880	.876	.882	.900	.891	.875	.861	.860	.895	.869	.884	.880	.905	.889
AMH AE muscle rigidity	12	.993	.997	.996	.998	.992	.997	.958	.997	.990	.997	.994	.998	.991	.997	.996	.997	.991	.998	.997	.998
AMH AE musculoskeletal pain	15	.908	.939	.889	.890	.885	.913	.913	.917	.927	.938	.896	.918	.942	.969	.908	.897	.937	.958	.856	.842
AMH AE myalgia	111	.695	.723	.655	.637	.690	.737	.657	.680	.725	.756	.710	.696	.710	.714	.701	.658	.703	.734	.634	.672
AMH AE mydriasis	20	.963	.954	.958	.958	.962	.907	.958	.957	.957	.894	.958	.958	.960	.912	.957	.958	.960	.883	.917	.956
AMH AE myelosuppression	37	.963	.959	.934	.974	.963	.979	.927	.974	.943	.972	.951	.946	.964	.980	.942	.976	.965	.981	.949	.977
AMH AE myocardial ischaemia	20	.887	.873	.874	.885	.837	.860	.835	.882	.840	.836	.874	.876	.869	.872	.850	.856	.901	.863	.897	.897
AMH AE myoclonus	14	.934	.965	.960	.957	.932	.949	.950	.951	.935	.963	.954	.964	.935	.955	.958	.957	.935	.952	.960	.959
AMH AE myopathy	32	.778	.882	.853	.871	.829	.873	.801	.812	.874	.927	.875	.874	.843	.864	.852	.860	.840	.888	.854	.863
AMH AE nasal congestion	26	.855	.865	.881	.883	.886	.818	.860	.861	.880	.920	.892	.889	.856	.889	.886	.885	.870	.881	.877	.875
AMH AE nausea	469	.643	.689	.654	.652	.659	.683	.663	.667	.676	.722	.667	.675	.636	.689	.656	.649	.638	.686	.661	.651
AMH AE nephropathy	12	.987	.992	.989	.988	.988	.993	.998	.983	.984	.992	.979	.971	.986	.993	.999	.999	.985	.993	.999	.989
AMH AE nephrotic syndrome	17	.901	.938	.948	.952	.878	.939	.938	.948	.917	.940	.944	.951	.880	.947	.951	.953	.902	.951	.957	.958

(Continue on next page)

Table A3. Cross-validation results by algorithms and drug characteristics (cont'd)

		AUC (method / algorithm)																			
		cdf				*ctf*				*cdf-icdf*				Stemming				Drug synonyms			
Characteristic name	n	NB	IBk	SVL	SVR	NB	IBk	SVL	SVR	NB	IBk	SVL	SVR	NB	IBk	SVL	SVR	NB	IBk	SVL	SVR
AMH AE nephrotoxicity	18	.808	.909	.919	.921	.896	.959	.979	.979	.909	.881	.935	.936	.783	.893	.904	.905	.746	.892	.865	.866
AMH AE nervousness	18	.644	.799	.464	.549	.562	.780	.442	.542	.788	.764	.753	.736	.659	.795	.558	.580	.623	.783	.470	.538
AMH AE neuroleptic malignant syndrome	13	.933	.988	.944	.979	.961	.987	.714	.851	.981	.986	.921	.959	.941	.984	.819	.970	.938	.977	.746	.976
AMH AE neuropathy	13	.823	.810	.810	.828	.808	.796	.769	.819	.813	.771	.796	.802	.767	.835	.770	.833	.816	.873	.880	.909
AMH AE neurotoxicity	30	.891	.940	.783	.799	.898	.938	.828	.856	.938	.950	.948	.938	.901	.936	.840	.813	.878	.923	.824	.869
AMH AE neutropenia	74	.791	.803	.721	.672	.772	.831	.691	.681	.803	.829	.772	.758	.789	.815	.656	.590	.764	.810	.699	.689
AMH AE nightmares	30	.920	.932	.946	.940	.920	.931	.914	.942	.912	.920	.905	.907	.909	.931	.930	.903	.905	.920	.941	.931
AMH AE ocular irritation	16	.903	.942	.880	.919	.888	.941	.879	.927	.909	.945	.848	.876	.865	.910	.830	.873	.889	.945	.864	.858
AMH AE oedema	51	.580	.712	.567	.675	.610	.709	.655	.671	.734	.783	.700	.702	.630	.726	.569	.684	.651	.706	.641	.697
AMH AE oesophageal ulceration	14	.992	.998	.999	.994	.991	.999	.999	.999	.991	.998	.998	.998	.993	.999	.999	.998	.992	.999	.999	.999
AMH AE oesophagitis	19	.907	.919	.833	.826	.878	.904	.720	.740	.917	.918	.894	.904	.899	.922	.699	.716	.911	.935	.739	.749
AMH AE oral mucositis	38	.974	.979	.957	.982	.971	.984	.966	.983	.953	.959	.949	.959	.971	.982	.964	.981	.971	.979	.937	.980
AMH AE orthostatic hypotension	77	.908	.925	.893	.906	.883	.916	.786	.813	.902	.923	.829	.838	.898	.901	.859	.862	.902	.921	.873	.890
AMH AE osteoporosis	15	.806	.820	.817	.822	.774	.854	.829	.826	.925	.910	.933	.933	.765	.829	.775	.755	.811	.890	.858	.822
AMH AE pain	61	.721	.680	.687	.732	.680	.673	.698	.740	.785	.747	.790	.791	.745	.770	.764	.748	.726	.717	.722	.720
AMH AE palpitations	68	.784	.785	.692	.649	.760	.748	.640	.633	.794	.839	.730	.755	.785	.793	.697	.699	.801	.799	.653	.633
AMH AE pancreatitis	83	.750	.748	.719	.718	.750	.732	.731	.725	.754	.795	.759	.756	.742	.760	.720	.729	.732	.733	.699	.707
AMH AE pancytopenia	18	.804	.843	.852	.853	.748	.814	.643	.664	.881	.803	.812	.819	.774	.791	.677	.756	.852	.808	.781	.809
AMH AE papillary necrosis	13	.993	.999	.999	.999	.991	.999	.999	.999	.991	.998	.998	.998	.993	.999	.999	.999	.992	.999	.999	.999
AMH AE paraesthesia	85	.772	.768	.728	.726	.739	.725	.724	.689	.728	.760	.682	.676	.720	.726	.703	.706	.745	.732	.736	.732
AMH AE paralytic ileus	12	.961	.919	.920	.940	.840	.947	.955	.957	.947	.928	.953	.954	.952	.913	.854	.861	.949	.919	.810	.834
AMH AE parkinsonism	20	.861	.838	.580	.751	.874	.883	.636	.721	.896	.938	.830	.840	.892	.908	.662	.773	.836	.912	.704	.741
AMH AE peripheral neuropathy	43	.732	.815	.589	.648	.718	.777	.538	.672	.783	.840	.557	.653	.741	.836	.549	.651	.747	.820	.557	.663
AMH AE peripheral oedema	65	.760	.768	.756	.759	.683	.714	.704	.704	.774	.776	.738	.745	.741	.737	.732	.729	.709	.738	.726	.728
AMH AE pharyngitis	22	.849	.868	.862	.847	.817	.855	.791	.794	.870	.897	.854	.850	.824	.854	.816	.813	.885	.881	.830	.842

(Continue on next page)

Table A3. Cross-validation results by algorithms and drug characteristics (cont'd)

		AUC (method / algorithm)																			
		cdf				*ctf*				*cdf-icdf*				Stemming				Drug synonyms			
Characteristic name	n	NB	IBk	SVL	SVR	NB	IBk	SVL	SVR	NB	IBk	SVL	SVR	NB	IBk	SVL	SVR	NB	IBk	SVL	SVR
AMH AE photosensitivity	57	.672	.752	.698	.694	.765	.785	.781	.772	.770	.794	.766	.764	.713	.746	.752	.737	.748	.774	.726	.710
AMH AE pneumonitis	15	.842	.907	.752	.709	.888	.897	.781	.748	.904	.883	.869	.854	.815	.885	.548	.662	.827	.864	.612	.696
AMH AE polyuria	16	.814	.756	.842	.838	.825	.766	.846	.852	.861	.890	.832	.836	.873	.840	.796	.813	.887	.849	.854	.847
AMH AE prolonged qt interval	18	.786	.860	.597	.709	.789	.888	.717	.811	.809	.843	.831	.847	.800	.867	.668	.725	.748	.850	.640	.648
AMH AE proteinuria	16	.817	.826	.775	.804	.804	.792	.770	.770	.787	.745	.797	.800	.775	.728	.806	.813	.781	.740	.789	.809
AMH AE psoriasis	12	.872	.945	.906	.946	.869	.956	.943	.957	.924	.941	.959	.949	.897	.919	.932	.906	.928	.934	.940	.938
AMH AE psychosis	22	.797	.830	.613	.620	.826	.840	.622	.606	.895	.750	.845	.848	.708	.767	.542	.471	.753	.788	.666	.642
AMH AE pulmonary fibrosis	12	.912	.918	.533	.583	.902	.915	.624	.693	.937	.916	.927	.919	.923	.914	.818	.775	.900	.940	.691	.620
AMH AE pulmonary oedema	15	.789	.854	.818	.812	.797	.831	.871	.875	.854	.873	.702	.691	.803	.839	.828	.850	.818	.838	.839	.832
AMH AE purpura	11	.792	.762	.809	.806	.761	.758	.822	.812	.906	.807	.855	.849	.839	.758	.773	.776	.778	.729	.750	.750
AMH AE rash	379	.644	.650	.683	.667	.649	.664	.682	.676	.669	.665	.675	.677	.644	.664	.676	.670	.647	.680	.675	.671
AMH AE rectal irritation	15	.940	.964	.947	.940	.930	.943	.921	.934	.936	.970	.919	.956	.931	.959	.945	.940	.940	.955	.949	.945
AMH AE renal failure	74	.745	.754	.749	.754	.703	.779	.612	.620	.803	.831	.836	.830	.728	.738	.753	.731	.760	.797	.776	.754
AMH AE respiratory arrest	12	.864	.863	.875	.869	.845	.862	.872	.863	.927	.957	.950	.968	.921	.962	.895	.953	.886	.910	.897	.907
AMH AE respiratory depression	20	.960	.983	.922	.977	.961	.979	.934	.952	.969	.977	.972	.976	.968	.978	.964	.970	.971	.978	.960	.963
AMH AE restlessness	11	.578	.593	.602	.584	.581	.721	.633	.714	.731	.623	.686	.702	.644	.721	.513	.566	.700	.726	.562	.688
AMH AE rhabdomyolysis	17	.845	.888	.759	.773	.891	.913	.853	.865	.894	.874	.809	.804	.863	.862	.860	.846	.899	.887	.880	.853
AMH AE rhinitis	14	.770	.776	.802	.810	.795	.796	.840	.844	.734	.793	.782	.782	.771	.771	.694	.708	.733	.771	.703	.692
AMH AE salt	13	.993	.999	1	.999	.991	.998	.999	.999	.991	.998	.999	.999	.993	.999	.999	.999	.993	.999	1	.999
AMH AE sedation	48	.924	.967	.965	.951	.924	.972	.942	.946	.943	.962	.940	.934	.911	.944	.935	.922	.919	.969	.949	.943
AMH AE seizures	103	.672	.684	.681	.666	.679	.706	.663	.645	.700	.729	.661	.652	.656	.721	.657	.646	.659	.715	.652	.652
AMH AE serum sickness	13	.919	.929	.941	.941	.913	.926	.941	.939	.932	.907	.945	.950	.912	.906	.925	.916	.856	.811	.860	.849
AMH AE serum sickness like syndrome	22	.987	.995	.993	.995	.986	.997	.994	.997	.985	.996	.997	.996	.987	.995	.994	.997	.986	.994	.996	.998
AMH AE sexual dysfunction	11	.908	.864	.866	.935	.916	.917	.935	.930	.886	.919	.968	.956	.884	.863	.919	.902	.865	.857	.875	.863
AMH AE siadh	23	.880	.901	.897	.906	.885	.907	.867	.886	.886	.908	.896	.899	.892	.864	.880	.902	.886	.929	.894	.893

(Continue on next page)

Table A3. Cross-validation results by algorithms and drug characteristics (cont'd)

		AUC (method / algorithm)																			
		cdf				*ctf*				*cdf-icdf*				Stemming				Drug synonyms			
Characteristic name	n	NB	IBk	SVL	SVR	NB	IBk	SVL	SVR	NB	IBk	SVL	SVR	NB	IBk	SVL	SVR	NB	IBk	SVL	SVR
AMH AE sinus tachycardia	7	.993	1	1	1	.993	.999	.978	.999	.995	.998	1	.999	.994	1	1	1	.993	1	.999	.999
AMH AE skin atrophy	10	.984	.994	.971	.996	.985	.992	.994	.996	.990	.997	.935	.996	.947	.981	.901	.985	.981	.997	.967	.997
AMH AE skin irritation	21	.916	.936	.935	.927	.898	.911	.925	.908	.906	.917	.911	.917	.878	.850	.892	.881	.860	.796	.894	.867
AMH AE skin pigmentation changes	41	.865	.856	.845	.844	.868	.879	.825	.823	.845	.858	.775	.796	.824	.849	.794	.773	.853	.858	.778	.780
AMH AE skin reactions	18	.744	.757	.694	.722	.734	.719	.563	.685	.817	.687	.789	.790	.721	.735	.628	.664	.835	.801	.713	.744
AMH AE somnolence	33	.734	.820	.612	.623	.765	.825	.712	.695	.771	.808	.694	.649	.761	.774	.800	.790	.756	.774	.671	.698
AMH AE sore throat	16	.898	.920	.849	.860	.338	.352	.348	.342	.838	.910	.851	.856	.887	.927	.875	.878	.851	.912	.867	.870
AMH AE spotting	10	.990	.993	.993	.993	.990	.995	.971	.996	.988	.997	.948	.987	.986	.994	.991	.991	.986	.994	.994	.993
AMH AE stevensjohnson syndrome	109	.735	.792	.739	.743	.738	.784	.749	.765	.765	.797	.773	.773	.744	.792	.771	.783	.750	.788	.765	.773
AMH AE stinging	21	.800	.737	.664	.672	.793	.728	.738	.722	.820	.797	.808	.814	.800	.703	.544	.577	.742	.764	.698	.713
AMH AE stinging on instillation	23	.919	.962	.961	.959	.904	.950	.962	.962	.957	.978	.948	.962	.898	.962	.936	.928	.911	.948	.930	.936
AMH AE stomatitis	19	.871	.870	.869	.869	.826	.904	.848	.865	.819	.875	.836	.837	.796	.858	.810	.814	.830	.889	.886	.885
AMH AE stroke	19	.847	.851	.784	.746	.866	.852	.723	.656	.855	.930	.811	.816	.830	.895	.682	.606	.844	.916	.722	.726
AMH AE subcutaneous tissue atrophy	10	.985	.995	.977	.995	.983	.991	.956	.994	.989	.997	.901	.995	.950	.983	.892	.986	.979	.994	.968	.995
AMH AE superinfections	25	.976	.991	.996	.993	.976	.993	.996	.994	.985	.993	.995	.995	.984	.991	.997	.995	.980	.991	.996	.994
AMH AE sweating	45	.773	.783	.738	.763	.722	.731	.686	.693	.779	.777	.756	.761	.754	.798	.781	.792	.748	.737	.716	.713
AMH AE syncope	20	.723	.707	.640	.656	.643	.721	.527	.630	.731	.736	.684	.697	.598	.726	.552	.604	.777	.761	.643	.687
AMH AE tachycardia	83	.786	.767	.696	.704	.771	.767	.696	.709	.770	.773	.720	.705	.809	.764	.722	.725	.820	.770	.679	.701
AMH AE taste disturbances	97	.709	.707	.692	.680	.683	.683	.644	.613	.713	.730	.660	.625	.677	.713	.559	.545	.717	.737	.624	.608
AMH AE tenderness	11	.936	.879	.942	.922	.937	.879	.892	.847	.874	.975	.818	.842	.885	.947	.862	.848	.893	.954	.900	.875
AMH AE thrombocytopenia	115	.752	.795	.616	.721	.736	.788	.624	.708	.768	.849	.738	.763	.726	.770	.589	.709	.732	.779	.592	.664
AMH AE thrombocytopenic purpura	11	.987	.996	.988	.989	.984	.991	.989	.984	.984	.993	.992	.990	.964	.964	.933	.947	.986	.983	.992	.990
AMH AE thrombophlebitis	31	.735	.746	.812	.812	.731	.800	.781	.789	.813	.684	.663	.697	.702	.760	.709	.713	.623	.653	.634	.600
AMH AE tinnitus	28	.791	.776	.794	.778	.811	.754	.792	.811	.814	.809	.794	.793	.762	.775	.795	.766	.780	.788	.813	.781
AMH AE torsades de pointes	11	.901	.901	.856	.848	.887	.870	.839	.849	.888	.831	.873	.872	.841	.859	.841	.845	.905	.863	.869	.894

(Continue on next page)

Table A3. Cross-validation results by algorithms and drug characteristics (cont'd)

		AUC (method / algorithm)																			
		cdf				*ctf*				*cdf-icdf*				Stemming				Drug synonyms			
Characteristic name	n	NB	IBk	SVL	SVR	NB	IBk	SVL	SVR	NB	IBk	SVL	SVR	NB	IBk	SVL	SVR	NB	IBk	SVL	SVR
AMH AE toxic epidermal necrolysis	83	.740	.818	.791	.783	.718	.801	.760	.759	.787	.837	.832	.827	.730	.828	.798	.801	.733	.815	.799	.800
AMH AE tremor	62	.817	.829	.702	.716	.810	.792	.683	.715	.815	.799	.705	.733	.807	.822	.690	.722	.813	.782	.693	.770
AMH AE urinary retention	51	.856	.853	.700	.704	.819	.870	.646	.670	.850	.879	.793	.787	.863	.847	.782	.805	.867	.889	.746	.775
AMH AE urticaria	98	.652	.655	.621	.628	.663	.675	.658	.634	.688	.677	.666	.675	.666	.613	.645	.635	.655	.675	.577	.590
AMH AE vasculitis	13	.796	.744	.732	.725	.798	.776	.725	.720	.691	.712	.717	.712	.827	.760	.836	.831	.796	.744	.734	.736
AMH AE vertigo	31	.700	.642	.637	.651	.725	.684	.563	.551	.748	.715	.624	.630	.755	.640	.644	.655	.736	.694	.665	.674
AMH AE visual disturbances	21	.785	.785	.851	.849	.804	.772	.797	.800	.761	.735	.797	.806	.804	.823	.825	.834	.774	.721	.790	.815
AMH AE vomiting	352	.677	.748	.693	.706	.687	.735	.699	.700	.707	.727	.703	.711	.674	.721	.685	.695	.663	.742	.687	.689
AMH AE vte	25	.800	.831	.658	.683	.779	.796	.676	.670	.808	.880	.721	.725	.799	.847	.760	.756	.765	.839	.782	.749
AMH AE weakness	71	.760	.766	.712	.716	.729	.779	.701	.703	.761	.757	.716	.688	.740	.727	.663	.649	.720	.717	.602	.610
AMH AE weight changes	20	.951	.929	.931	.936	.941	.925	.926	.932	.909	.990	.893	.898	.918	.958	.890	.897	.927	.971	.896	.901
AMH AE weight gain	65	.829	.842	.830	.844	.832	.855	.860	.872	.844	.905	.859	.866	.795	.823	.780	.779	.800	.794	.792	.797
AMH AE weight loss	12	.790	.746	.789	.822	.740	.726	.755	.750	.860	.821	.907	.906	.817	.811	.834	.830	.764	.827	.855	.852
AMH drug class allergy and anaphylaxis	11	.952	.971	.978	.979	.950	.967	.969	.968	.964	.978	.978	.981	.951	.969	.971	.975	.946	.965	.916	.974
AMH drug class anaesthetics	29	.977	.989	.993	.991	.974	.990	.991	.990	.980	.988	.992	.993	.978	.991	.993	.992	.976	.990	.993	.992
AMH drug class analgesics	12	.974	.990	.994	.994	.974	.990	.995	.994	.972	.988	.989	.992	.971	.985	.991	.991	.956	.980	.984	.986
AMH drug class anti infectives	135	.974	.979	.985	.984	.976	.984	.986	.983	.975	.983	.985	.982	.973	.983	.986	.984	.968	.981	.985	.980
AMH drug class antidotes and antivenoms	28	.842	.842	.910	.875	.882	.883	.918	.905	.863	.899	.915	.872	.816	.779	.914	.884	.844	.833	.930	.896
AMH drug class blood and electrolytes	37	.872	.886	.911	.912	.895	.927	.904	.926	.931	.947	.962	.941	.883	.888	.915	.909	.877	.885	.889	.899
AMH drug class cardiovascular drugs	89	.918	.932	.941	.942	.911	.931	.933	.934	.958	.972	.963	.963	.916	.913	.935	.936	.915	.930	.935	.937
AMH drug class dermatological drugs	64	.911	.933	.944	.944	.906	.926	.940	.940	.921	.949	.929	.933	.872	.890	.903	.907	.875	.888	.908	.918
AMH drug class ear nose and throat drugs	32	.805	.796	.743	.811	.786	.803	.811	.818	.795	.854	.807	.828	.837	.780	.878	.867	.716	.739	.778	.782
AMH drug class endocrine drugs	54	.939	.963	.890	.858	.903	.903	.843	.840	.953	.972	.963	.958	.910	.883	.888	.877	.936	.883	.903	.892
AMH drug class eye drugs	44	.908	.974	.965	.970	.896	.980	.973	.971	.956	.967	.982	.979	.922	.954	.970	.957	.905	.870	.956	.942
AMH drug class gastrointestinal drugs	65	.869	.891	.896	.901	.851	.875	.868	.880	.900	.872	.891	.893	.855	.853	.879	.876	.852	.824	.872	.875

(Continue on next page)

Table A3. Cross-validation results by algorithms and drug characteristics (cont'd)

		AUC (method / algorithm)																			
		cdf				*ctf*				*cdf-icdf*				Stemming				Drug synonyms			
Characteristic name	n	NB	IBk	SVL	SVR	NB	IBk	SVL	SVR	NB	IBk	SVL	SVR	NB	IBk	SVL	SVR	NB	IBk	SVL	SVR
AMH drug class genitourinary drugs	15	.990	.995	.993	.998	.988	.996	.988	.997	.990	.980	.984	.993	.989	.988	.994	.992	.989	.992	.983	.997
AMH drug class immunomodulators and antineoplastics	104	.958	.982	.984	.982	.960	.979	.985	.983	.959	.977	.974	.975	.954	.978	.982	.981	.964	.986	.986	.985
AMH drug class minor 5 aminosalicylates	4	.997	1	1	1	.997	1	.950	.963	.998	1	1	1	.998	1	1	1	.998	1	1	1
AMH drug class minor 5ht 3 antagonists	4	.995	1	.999	1	.149	.150	.150	.150	.997	.999	.998	.998	.997	1	1	1	.997	.999	.999	.999
AMH drug class minor ace inhibitors	8	.994	1	1	1	.994	1	1	1	.996	1	1	1	.994	1	1	1	.993	1	1	1
AMH drug class minor aldosterone antagonists	2	.500	1	.999	.999	.500	.999	.999	.998	.550	.999	.999	.999	.525	.998	.997	.997	.625	.999	.999	1
AMH drug class minor alkylating agents	12	.890	.896	.879	.894	.989	.997	.990	.979	.990	.996	.999	.999	.990	.996	.983	.979	.989	.995	.994	.984
AMH drug class minor alpha 2 agonists	2	.500	.996	.997	.997	.500	.996	.997	.996	.500	.979	.996	.996	.500	.997	.996	.997	.500	.991	.997	.998
AMH drug class minor alpha 2 and imidazoline agonists	2	.500	.728	.727	.727	.350	.565	.568	.567	.550	.999	.996	.995	.649	1	.997	.997	.499	.997	.998	.997
AMH drug class minor aminoglycosides	4	.249	.250	.250	.250	.183	.245	.244	.246	.998	.999	.995	.985	.997	.999	.994	.998	.996	.998	.994	.998
AMH drug class minor aminoglycosides eye	3	.823	.810	.813	.813	.821	.809	.811	.810	.894	.979	.894	.878	.874	.982	.977	.946	.891	.980	.951	.951
AMH drug class minor androgenetic alopecia	2	.500	.997	.991	.991	.250	.383	.381	.382	.573	.998	.995	.995	.617	.991	.996	.996	.596	.991	.991	.991
AMH drug class minor androgens	1	-	-	-	-	-	-	-	-	-	-	-	-	-	-	-	-	-	-	-	-
AMH drug class minor antacids	3	.965	.999	.929	.931	.649	.663	.663	.647	.845	.979	.991	.993	.912	.999	.995	.995	.876	.991	.992	.989
AMH drug class minor anthracyclines	5	.793	.800	.800	.800	.919	.960	.960	.960	.991	.998	.957	.977	.991	.999	.999	.999	.993	.998	.988	.998
AMH drug class minor anti androgens	4	.996	1	1	1	.996	.999	.999	1	.998	1	.999	1	.996	.999	.999	.999	.996	1	1	1
AMH drug class minor antiarrhythmics	11	.968	.984	.991	.994	.967	.987	.990	.990	.985	.989	.996	.996	.970	.991	.980	.993	.970	.987	.984	.991
AMH drug class minor antibacterials ear	4	.956	.981	.692	.833	.866	.960	.602	.842	.855	.958	.849	.833	.960	.972	.807	.866	.949	.970	.713	.732
AMH drug class minor antibacterials skin	6	.924	.968	.971	.932	.907	.959	.960	.933	.934	.974	.925	.925	.964	.961	.970	.970	.916	.962	.966	.967
AMH drug class minor anticholinergics	4	.996	1	1	1	.993	1	1	1	.993	1	.949	1	.996	1	1	1	.949	.999	.935	.960
AMH drug class minor anticholinergics anaesthesia	1	-	-	-	-	-	-	-	-	-	-	-	-	-	-	-	-	-	-	-	-
AMH drug class minor anticholinergics eye	4	.926	.989	.994	.994	.927	.992	.993	.994	.925	.989	.925	.990	.923	.989	.988	.977	.954	.998	.997	.997
AMH drug class minor anticholinergics genitourinary	5	.792	.800	.800	.800	.796	.800	.800	.800	.798	.800	.800	.800	.794	.800	.800	.800	.794	.800	.800	.800
AMH drug class minor anticholinergics inhaled	2	.500	.999	.997	.996	.250	.499	.496	.497	.500	.996	.995	.994	.500	.998	.996	.996	.524	.998	.996	.996
AMH drug class minor anticholinesterases in alzheimers disease	3	1	1	1	1	0	0	0	0	.999	1	1	1	1	1	.999	1	1	1	.999	1

(Continue on next page)

Table A3. Cross-validation results by algorithms and drug characteristics (cont'd)

		AUC (method / algorithm)																			
		cdf				*ctf*				*cdf-icdf*				Stemming				Drug synonyms			
Characteristic name	n	NB	IBk	SVL	SVR	NB	IBk	SVL	SVR	NB	IBk	SVL	SVR	NB	IBk	SVL	SVR	NB	IBk	SVL	SVR
AMH drug class minor anticholinesterases in myasthenia gravis	2	.500	.984	.983	.982	.500	.919	.917	.916	.500	.972	.961	.960	.522	.936	.931	.930	.647	.995	.991	.991
AMH drug class minor antidiuretic hormone agonists and antagonists	4	.994	.999	.999	.998	.990	.999	.998	.998	.995	.999	.936	.974	.996	.999	.999	.999	.997	.999	.999	.999
AMH drug class minor antidotes	15	.902	.964	.960	.965	.954	.976	.984	.984	.954	.822	.947	.939	.882	.950	.944	.939	.868	.956	.937	.941
AMH drug class minor antihistamines antiemetic	2	.500	.690	.686	.686	.500	.694	.694	.693	.500	.982	.994	.993	.546	.919	.928	.926	.499	.769	.768	.767
AMH drug class minor antihistamines eye	4	.994	1	1	1	.995	1	1	1	.996	.999	.998	.998	.995	1	1	.999	.993	.999	.999	1
AMH drug class minor antihistamines intranasal	2	.500	.921	.909	.886	.500	.930	.950	.949	.500	.971	.962	.960	.500	.941	.965	.965	.500	.760	.752	.752
AMH drug class minor antimalarials	8	.998	.999	.917	.999	.997	.999	.943	.999	.998	.998	.973	.999	.998	.999	.968	.992	.998	.999	.962	.999
AMH drug class minor antimetabolites	12	.904	.913	.918	.919	.989	.996	.999	.999	.986	.995	1	1	.984	.987	.999	.999	.983	.986	.993	.983
AMH drug class minor antineoplastic antibodies	6	.989	.999	1	1	.990	1	1	1	.992	1	.999	1	.990	.997	1	.999	.991	.998	.983	.999
AMH drug class minor antipsychotics	18	.992	.997	.990	.999	.991	.996	.999	.998	.990	.995	.982	.996	.984	.994	.999	.996	.992	.995	.999	.998
AMH drug class minor antiseptics ear	3	.677	.689	.692	.690	.411	.457	.461	.460	.947	.996	.996	.996	.911	.994	.993	.995	.947	.997	.996	.997
AMH drug class minor antithyroid drugs	2	.250	.500	.500	.500	.250	.500	.500	.499	.250	.500	.500	.500	.300	.500	.500	.500	.275	.500	.500	.500
AMH drug class minor antivirals eye	1	-	-	-	-	-	-	-	-	-	-	-	-	-	-	-	-	-	-	-	-
AMH drug class minor antivirals skin	3	.552	.520	.519	.519	.273	.228	.228	.234	.357	.299	.332	.341	.097	.093	.096	.096	.355	.318	.341	.341
AMH drug class minor aromatase inhibitors	3	.999	1	1	1	.799	.800	.800	.800	.999	1	1	1	.999	1	1	1	1	1	1	1
AMH drug class minor attention deficit hyperactivity disorder	3	.998	.999	.999	.983	.798	.798	.798	.749	.999	.999	.966	.950	.999	.999	.999	.933	.983	.999	.998	.999
AMH drug class minor azoles	6	.993	.997	.998	.997	.994	.998	.981	.990	.996	.999	.999	.991	.994	.998	.955	.998	.995	.998	.948	.965
AMH drug class minor azoles skin	5	0	0	0	0	.794	.799	.798	.798	.993	.998	.998	.998	.994	.999	.998	.998	.994	.999	.998	.998
AMH drug class minor azoles vaginal	3	.827	.808	.789	.807	.830	.804	.734	.788	.746	.799	.799	.798	.743	.794	.793	.796	.745	.762	.763	.763
AMH drug class minor barbiturates	2	.500	.985	.979	.978	.250	.487	.476	.473	.498	.832	.820	.818	.574	.979	.956	.955	.497	.775	.762	.760
AMH drug class minor bcg	5	.766	.802	.769	.780	.773	.935	.779	.772	.747	.718	.515	.554	.747	.761	.747	.745	.750	.782	.767	.767
AMH drug class minor benzimidazoles	2	.600	1	.997	.998	.500	.999	.998	.999	.850	1	.998	1	.925	1	.998	.999	.900	1	.999	1
AMH drug class minor benzodiazepines	10	0	0	0	0	0	0	0	0	.796	.799	.800	.799	.797	.799	.720	.800	0	0	0	0
AMH drug class minor benzodiazepines epilepsy	5	0	0	0	0	0	0	0	0	.793	.798	.796	.795	.792	.796	.796	.755	.794	.795	.796	.785
AMH drug class minor beta 2 agonists	4	.990	.998	.996	.997	.991	.999	.999	.999	.994	.999	.996	.984	.995	.999	.999	1	.992	.997	.997	.960

(Continue on next page)

Table A3. Cross-validation results by algorithms and drug characteristics (cont'd)

		AUC (method / algorithm)																			
		cdf				*ctf*				*cdf-icdf*				Stemming				Drug synonyms			
Characteristic name	n	NB	IBk	SVL	SVR	NB	IBk	SVL	SVR	NB	IBk	SVL	SVR	NB	IBk	SVL	SVR	NB	IBk	SVL	SVR
AMH drug class minor beta blockers	8	.992	.999	.999	.969	.992	.999	1	.994	.994	.998	.973	.967	.993	.998	.999	.974	.994	.997	.999	.975
AMH drug class minor beta blockers eye	2	.400	.512	.514	.513	.250	.490	.490	.490	.425	.500	.499	.499	.399	.499	.499	.499	.374	.499	.496	.497
AMH drug class minor bile acid binding resins	2	0	0	0	0	.250	.378	.364	.369	.500	1	1	1	.500	1	.999	.999	.525	.995	.993	.993
AMH drug class minor bipolar disorder	1	-	-	-	-	-	-	-	-	-	-	-	-	-	-	-	-	-	-	-	-
AMH drug class minor bisphosphonates	8	.995	1	1	1	.993	1	1	1	.997	1	1	1	.996	1	1	1	.994	1	1	1
AMH drug class minor calcineurin inhibitors	2	.600	.998	.994	.994	0	0	0	0	.697	.997	.995	.995	.823	.997	.995	.995	.774	.999	.998	.998
AMH drug class minor calcium channel blockers	7	0	0	0	0	0	0	0	0	0	0	0	0	0	0	0	0	0	0	0	0
AMH drug class minor carbapenems	3	.979	1	1	1	.232	.266	.266	.266	.964	1	1	1	.980	1	1	1	.997	.999	.999	.999
AMH drug class minor carbonic anhydrase inhibitors	2	.550	1	1	1	.500	1	.999	.998	.525	1	1	1	.500	1	1	1	.650	1	1	1
AMH drug class minor cephalosporins	10	.992	.999	.934	.974	.993	.999	1	.984	.992	.999	1	.999	.994	.999	.956	.989	.993	.997	.973	.993
AMH drug class minor cerumenolytics	4	.386	.392	.373	.358	.621	.400	.485	.525	.628	.533	.526	.500	.542	.351	.397	.359	.637	.351	.547	.449
AMH drug class minor chronic hepatitis c	5	.992	.998	.946	.939	.993	.999	.926	.939	.997	.998	.949	.949	.993	.999	.989	1	.991	.998	.989	.980
AMH drug class minor colony stimulating factors	3	.909	1	1	1	.823	.999	.950	1	.832	.974	.991	.987	.911	1	1	1	.893	1	1	1
AMH drug class minor combined oral contraceptives	8	.911	.810	.908	.909	.912	.713	.914	.893	.832	.980	.654	.751	.828	.938	.777	.767	.823	.947	.781	.768
AMH drug class minor corticosteroids	9	.983	.995	.943	.965	.984	.994	.957	.966	.990	.999	.991	.970	.947	.984	.963	.988	.988	.998	.983	.994
AMH drug class minor corticosteroids eye	4	.859	.853	.861	.850	.737	.733	.617	.655	.938	.987	.875	.838	.688	.954	.844	.840	.958	.991	.929	.955
AMH drug class minor corticosteroids gastrointestinal	3	.736	.911	.917	.898	.677	.755	.739	.737	.842	.981	.890	.857	.583	.911	.574	.549	.924	.989	.970	.923
AMH drug class minor corticosteroids inhaled	4	.995	.999	.998	.999	.995	1	1	.999	.996	.999	.998	.998	.996	.998	.998	.998	.996	1	.998	.998
AMH drug class minor corticosteroids intranasal	5	.874	.879	.879	.879	.992	1	1	.999	.992	1	.999	.999	.994	1	1	.999	.993	1	1	.999
AMH drug class minor corticosteroids skin	7	.989	.992	.976	.962	.987	.989	.975	.969	.989	.987	.962	.996	.948	.974	.978	.976	.985	.988	.974	.959
AMH drug class minor corticosteroids with anti infectives ear	4	.770	.571	.663	.805	.768	.634	.346	.803	.486	.924	.373	.402	.770	.455	.717	.703	.395	.700	.340	.319
AMH drug class minor cromolyns	2	.500	1	.999	.999	.500	.999	.998	.998	.625	1	1	1	.775	1	.999	1	.250	.500	.499	.499
AMH drug class minor cytoprotective agents	2	.500	.973	.986	.986	.500	.975	.966	.962	.500	.996	.996	.996	.600	.990	.990	.988	.650	.991	.993	.993
AMH drug class minor depolarising neuromuscular blockers	1	-	-	-	-	-	-	-	-	-	-	-	-	-	-	-	-	-	-	-	-
AMH drug class minor dopamine agonists	3	.995	1	.999	.999	.997	1	1	.999	1	1	1	1	.997	1	1	1	.996	1	1	1

(Continue on next page)

Table A3. Cross-validation results by algorithms and drug characteristics (cont'd)

		AUC (method / algorithm)																			
		cdf				*ctf*				*cdf-icdf*				Stemming				Drug synonyms			
Characteristic name	n	NB	IBk	SVL	SVR	NB	IBk	SVL	SVR	NB	IBk	SVL	SVR	NB	IBk	SVL	SVR	NB	IBk	SVL	SVR
AMH drug class minor dopamine agonists parkinsonism	6	.993	.995	.920	.995	.099	.099	.091	.099	.991	.994	.980	.954	.992	.993	.954	.987	.993	.997	.988	.989
AMH drug class minor dopamine antagonists antiemetic	5	.987	.992	.967	.977	.934	.983	.973	.945	.927	.974	.921	.929	.927	.973	.986	.928	.985	.995	.968	.977
AMH drug class minor drugs affecting gastrointestinal motility	4	.983	.989	.940	.905	.969	.987	.948	.977	.968	.993	.934	.935	.988	.996	.993	.982	.989	.995	.980	.996
AMH drug class minor drugs for actinic keratoses	3	.476	.661	.545	.609	.676	.931	.646	.582	.654	.921	.755	.747	.690	.920	.732	.665	.649	.900	.834	.840
AMH drug class minor drugs for multiple sclerosis	3	.999	.999	.983	.999	.996	.999	.949	.949	.982	.999	.999	.999	.964	.999	.996	.999	.998	1	.983	1
AMH drug class minor drugs for peripheral vascular disease	2	.500	.318	.358	.356	.150	.058	.058	.058	.500	1	.975	.986	.500	1	1	1	.500	.994	.992	.991
AMH drug class minor dry eye syndrome	1	-	-	-	-	-	-	-	-	-	-	-	-	-	-	-	-	-	-	-	-
AMH drug class minor endometriosis	2	.500	.705	.705	.705	.500	.689	.689	.689	.525	.999	.999	.999	.500	.973	.996	.996	.350	.695	.694	.694
AMH drug class minor endothelin antagonists	2	.500	.691	.691	.691	.250	.498	.498	.498	.500	.596	.596	.596	.525	.725	.724	.724	.500	.720	.720	.720
AMH drug class minor ergot alkaloids	2	.450	.894	.894	.894	.500	.997	.992	.993	.524	1	.997	.997	.647	.999	.997	.998	.750	.999	.997	.997
AMH drug class minor erythropoietin agonists	3	1	1	1	1	1	1	1	1	1	1	1	1	1	1	1	1	1	1	1	1
AMH drug class minor fibrates	2	.500	1	.999	.999	.250	.500	.499	.499	.775	1	1	1	1	1	.998	.999	1	1	.999	.999
AMH drug class minor gastrointestinal decontamination	2	.500	.675	.641	.640	.250	.477	.483	.482	.499	.557	.642	.640	.495	.581	.575	.575	.495	.654	.636	.632
AMH drug class minor glycopeptides	2	.250	.500	.499	.499	.250	.499	.497	.497	.900	1	1	1	.850	.997	.995	.995	.875	.996	.996	.996
AMH drug class minor glycoprotein iib iiia inhibitors	3	.999	1	1	1	.999	1	1	1	.999	1	1	1	.999	1	1	1	.999	1	1	1
AMH drug class minor gold salts	2	.500	1	.999	.998	.500	.999	.995	.994	.500	1	1	1	.625	1	.999	.999	.675	1	1	1
AMH drug class minor gonadotrophin releasing hormone agonists	2	.500	.977	.986	.985	.500	.736	.732	.729	.500	.958	.983	.982	.500	.996	.994	.994	.200	.399	.397	.397
AMH drug class minor gonadotrophin releasing hormone agonists oncology	3	.998	.999	.999	.999	.998	1	.999	1	.998	1	1	1	.999	.999	.999	.999	.998	.999	1	.999
AMH drug class minor gout	3	.995	.994	.990	.993	.580	.662	.662	.661	.548	.664	.643	.646	.562	.661	.608	.593	.615	.660	.610	.643
AMH drug class minor growth hormone	1	-	-	-	-	-	-	-	-	-	-	-	-	-	-	-	-	-	-	-	-

(Continue on next page)

Table A3. Cross-validation results by algorithms and drug characteristics (cont'd)

		AUC (method / algorithm)																			
		cdf				*ctf*				*cdf-icdf*				Stemming				Drug synonyms			
Characteristic name	n	NB	IBk	SVL	SVR	NB	IBk	SVL	SVR	NB	IBk	SVL	SVR	NB	IBk	SVL	SVR	NB	IBk	SVL	SVR
AMH drug class minor guanine analogues	5	.996	.996	.937	.956	.996	.996	.967	.947	.996	.996	.946	.977	.997	.996	.978	.986	.996	.998	.968	.936
AMH drug class minor h 2 antagonists	4	.547	.550	.550	.550	.547	.550	.550	.550	.996	1	.999	1	.995	1	1	1	.995	1	1	1
AMH drug class minor head lice	5	.852	.690	.821	.831	.903	1	1	1	.845	.793	.852	.851	.854	.749	.844	.862	.872	.668	.881	.882
AMH drug class minor heparins	4	.984	.997	.997	.973	.995	.999	.999	.999	.994	.998	.998	.999	.997	.999	.999	.999	.997	.998	.999	.974
AMH drug class minor hormone replacement therapy	5	.989	.999	.978	.999	.990	.998	.946	.988	.990	.998	.978	.929	.991	.998	.999	.999	.990	1	1	.999
AMH drug class minor hypoglycaemia	1	-	-	-	-	-	-	-	-	-	-	-	-	-	-	-	-	-	-	-	-
AMH drug class minor immunosuppressant antibodies	3	.995	1	1	1	.997	1	.999	.999	.997	1	.999	.999	.996	1	.999	.999	.996	1	.999	.999
AMH drug class minor immunosuppressants psoriasis	3	.629	.792	.765	.765	.221	.285	.253	.267	.579	.924	.782	.768	.735	.947	.686	.673	.648	.950	.650	.649
AMH drug class minor immunosuppressants rheumatoid arthritis	7	.912	.978	.989	.987	.885	.979	.923	.857	.983	.981	.986	.986	.968	.984	.977	.990	.963	.982	.985	.984
AMH drug class minor inhaled anaesthetics	5	.992	1	1	1	.159	.160	.160	.160	.994	1	1	1	.989	1	1	1	.991	1	1	1
AMH drug class minor interferons	2	.500	.936	.910	.902	0	0	0	0	.499	.928	.906	.927	.500	.956	.895	.934	.500	.947	.898	.919
AMH drug class minor intranasal decongestants	5	.939	.988	.945	.992	.938	.990	.945	.980	.977	.989	.731	.718	.935	.983	.936	.942	.934	.984	.896	.943
AMH drug class minor intrauterine devices	2	.275	.500	.500	.500	.500	1	.999	.999	.875	1	1	1	.850	1	1	1	.800	1	1	1
AMH drug class minor iv general anaesthetics	4	.979	.996	.983	.971	.321	.368	.370	.370	.963	.990	.932	.918	.952	.993	.959	.958	.936	.992	.957	.944
AMH drug class minor less sedating antihistamines	5	.996	1	1	1	.996	1	1	1	.998	1	1	1	.997	1	1	1	.997	1	1	1
AMH drug class minor leukotriene receptor antagonists	2	.550	1	1	1	.250	.500	.499	.499	.900	1	1	1	.825	1	1	1	.800	1	1	1
AMH drug class minor lincosamides	2	.500	.988	.975	.970	.250	.272	.273	.273	.598	.992	.984	.983	.573	.894	.885	.885	.648	.994	.991	.991
AMH drug class minor local anaesthetics	8	.952	.984	.984	.962	.952	.984	.963	.962	.952	.984	.972	.963	.944	.975	.969	.975	.943	.975	.949	.967
AMH drug class minor local anaesthetics eye	3	.996	1	.999	.999	.997	1	1	1	.915	1	1	1	.998	.999	1	1	.965	1	1	.999
AMH drug class minor long term treatment for alcoholism	3	.997	.999	1	1	.995	.999	1	.999	.998	1	.999	1	.996	.999	1	1	.997	.999	.999	.999
AMH drug class minor loop diuretics	3	.665	.664	.664	.665	0	0	0	0	.998	.999	.999	.999	.997	.998	.998	.998	.864	.866	.866	.866
AMH drug class minor macrolides	4	.996	1	.999	.999	.797	.799	.799	.799	.996	1	1	1	.996	.999	1	1	.995	.999	.999	1
AMH drug class minor monoamine oxidase inhibitors	2	.500	.999	.998	.998	.500	1	.997	.997	.500	1	.998	.998	.575	1	.997	.998	.675	.999	.996	.996
AMH drug class minor mucolytics	3	.576	.666	.667	.667	.977	.999	1	1	.831	.994	.996	.999	.892	.999	1	1	.899	.999	1	1
AMH drug class minor mycobacterium avium complex	8	.997	.998	.992	.998	.997	.998	.998	.998	.993	.998	.967	.986	.976	.997	.985	.997	.964	.997	.998	.997

(Continue on next page)

Table A3. Cross-validation results by algorithms and drug characteristics (cont'd)

		AUC (method / algorithm)																			
		cdf				*ctf*				*cdf-icdf*				Stemming				Drug synonyms			
Characteristic name	n	NB	IBk	SVL	SVR	NB	IBk	SVL	SVR	NB	IBk	SVL	SVR	NB	IBk	SVL	SVR	NB	IBk	SVL	SVR
AMH drug class minor nasal staphylococcus aureus	1	-	-	-	-	-	-	-	-	-	-	-	-	-	-	-	-	-	-	-	-
AMH drug class minor neuraminidase inhibitors	2	.575	1	.999	.999	0	0	0	0	1	1	1	1	.925	1	.998	1	.950	1	1	1
AMH drug class minor nicotine dependence	3	.999	.999	.999	.999	.997	1	1	1	.999	1	1	1	.999	1	.999	.999	.999	1	1	1
AMH drug class minor nitrates	3	0	0	0	0	.467	.466	.450	.450	.583	.667	.667	.667	.632	.667	.667	.667	.999	1	1	1
AMH drug class minor nitroimidazoles	2	.500	.992	.995	.994	.250	.497	.496	.496	.725	1	.998	.998	.750	1	.999	.998	.850	1	.999	.999
AMH drug class minor non depolarising neuromuscular blockers	6	.995	1	1	1	.995	.999	1	1	.996	1	.999	1	.996	.999	1	1	.996	1	1	1
AMH drug class minor non nucleoside reverse transcriptase inhibitors	4	.994	1	1	1	.745	.750	.750	.750	.994	1	1	1	.993	1	1	1	.993	1	1	1
AMH drug class minor non opioid analgesics	2	.500	.691	.682	.682	.050	.097	.094	.094	.524	.889	.893	.893	.499	.894	.891	.891	.500	.971	.959	.959
AMH drug class minor nonselective alpha blockers	2	.500	.981	.975	.973	.250	.497	.490	.489	.550	.999	.997	.996	.725	.998	.997	.996	.798	1	.998	.998
AMH drug class minor nsaids	13	.993	.998	.999	.999	.991	.999	.993	.999	.991	.999	.998	.999	.993	.999	.999	.999	.993	.999	.999	.999
AMH drug class minor nsaids eye	3	.598	.598	.598	.598	.680	.793	.793	.793	.573	.664	.665	.664	.694	.797	.798	.798	.630	.663	.645	.647
AMH drug class minor nucleoside reverse transcriptase inhibitors	6	.929	.932	.932	.932	.994	.998	.998	.998	.828	.832	.833	.832	.826	.831	.789	.814	.827	.832	.833	.791
AMH drug class minor obesity	3	.832	.999	.999	.999	.250	.333	.333	.333	1	1	1	1	.948	.998	.998	.998	.832	1	1	1
AMH drug class minor opioid analgesics	10	.992	.996	.978	.994	.992	.995	.973	.974	.991	.998	.993	.999	.991	.994	.997	.998	.992	.996	.979	.999
AMH drug class minor opioid antidiarrhoeals	3	.857	.925	.921	.906	.512	.530	.529	.530	.902	.989	.988	.986	.895	.986	.979	.980	.908	.997	.996	.997
AMH drug class minor opioid cough suppressants	4	.991	1	1	1	.992	1	1	1	.990	.998	1	.997	.992	1	1	1	.993	1	1	1
AMH drug class minor opioid dependence	2	.500	1	.996	.996	.150	.297	.297	.297	.325	.500	.500	.500	.799	1	1	1	.700	1	.999	.999
AMH drug class minor opioids anaesthesia	2	.500	1	1	.999	.150	.300	.296	.296	.499	.992	.941	.965	.524	.994	.991	.991	.524	.995	.995	.995
AMH drug class minor oral decongestants	2	.300	.546	.515	.515	.500	.951	.937	.914	.400	.711	.757	.764	.400	.596	.595	.597	.250	.473	.469	.469
AMH drug class minor osmotic laxatives	5	.944	.967	.971	.990	.953	.954	.982	.990	.987	.991	.964	.985	.870	.939	.966	.974	.929	.976	.876	.980
AMH drug class minor oxytocic drugs	3	.996	1	.997	.999	.997	.999	.997	.982	.999	1	.983	1	.997	.999	.998	.999	.996	.998	.998	.999
AMH drug class minor penicillins	12	.986	.994	.994	.989	.986	.996	.995	.984	.983	.993	.968	.972	.985	.994	.999	1	.984	.992	.999	.989
AMH drug class minor perianal disorders	2	.500	.653	.668	.669	.500	.547	.556	.556	.500	.926	.900	.901	.500	.968	.975	.976	.500	.965	.981	.982
AMH drug class minor phosphate binders	4	.914	.993	.995	.993	.993	.996	.998	.997	.992	.998	.985	.948	.923	.993	.978	.933	.881	.991	.969	.957
AMH drug class minor phosphodiesterase 5 inhibitors	3	.999	1	.997	.998	.999	1	.998	.999	.999	1	.999	1	.999	1	.998	.999	.999	1	.999	1

(Continue on next page)

Table A3. Cross-validation results by algorithms and drug characteristics (cont'd)

		AUC (method / algorithm)																			
		cdf				*ctf*				*cdf-icdf*				Stemming				Drug synonyms			
Characteristic name	n	NB	IBk	SVL	SVR	NB	IBk	SVL	SVR	NB	IBk	SVL	SVR	NB	IBk	SVL	SVR	NB	IBk	SVL	SVR
AMH drug class minor platinum compounds	3	.147	.163	.163	.163	0	0	0	0	.666	.667	.667	.667	.666	.667	.667	.667	.665	.667	.667	.667
AMH drug class minor podophyllotoxins	2	.250	.496	.497	.496	.350	.685	.683	.682	.650	.996	.995	.994	.600	.995	.993	.992	.675	.995	.994	.994
AMH drug class minor potassium	1	-	-	-	-	-	-	-	-	-	-	-	-	-	-	-	-	-	-	-	-
AMH drug class minor potassium sparing diuretics	1	-	-	-	-	-	-	-	-	-	-	-	-	-	-	-	-	-	-	-	-
AMH drug class minor pre eclampsia and eclampsia	1	-	-	-	-	-	-	-	-	-	-	-	-	-	-	-	-	-	-	-	-
AMH drug class minor premature labour	2	.400	.505	.499	.499	.250	.318	.311	.311	.500	.826	.885	.882	.499	.653	.648	.651	.449	.667	.641	.657
AMH drug class minor progestogens	4	.929	.992	.960	.949	.644	.642	.642	.643	.983	.996	.997	.971	.995	.997	.997	.997	.995	.998	.997	.997
AMH drug class minor prostacyclins	3	.982	.999	.999	.999	.982	1	1	1	.965	1	.999	.999	.966	1	.999	.999	.949	1	.999	1
AMH drug class minor prostaglandin analogues eye	3	.666	.666	.666	.666	.933	.933	.933	.933	.666	.667	.667	.667	.666	.667	.667	.667	.666	.667	.667	.667
AMH drug class minor prostaglandins	4	.994	.998	.998	.999	.993	.999	.999	.999	.997	.999	1	1	.993	.999	.998	.999	.992	.997	.996	.997
AMH drug class minor protease inhibitors	8	.995	1	1	1	.994	1	1	1	.994	1	1	1	.994	1	1	1	.994	1	1	1
AMH drug class minor proton pump inhibitors	5	.756	.760	.760	.760	.318	.320	.320	.320	0	0	0	0	.794	.800	.799	.800	.159	.159	.160	.160
AMH drug class minor pulmonary surfactants	2	.500	1	.900	.875	.500	1	.947	.947	.500	.702	.999	.999	.625	1	1	1	.625	1	1	1
AMH drug class minor quinolones	3	.999	1	1	1	.999	.998	.998	.999	.999	1	1	1	.999	.999	.999	.999	.999	1	.999	.999
AMH drug class minor quinolones eye	2	.599	.998	.995	.995	.225	.399	.395	.395	.873	.997	.990	.990	.849	.998	.993	.993	.774	.995	.990	.990
AMH drug class minor retinoids oral	2	.500	.845	.857	.856	.250	.440	.436	.436	.598	.999	.996	.996	.624	.997	.992	.992	.550	.995	.991	.991
AMH drug class minor retinoids skin	4	.995	.997	.999	.999	.995	.997	.998	.998	.997	.998	.999	.999	.960	.998	.999	.999	.997	.999	.999	1
AMH drug class minor rifamycins	2	.500	.713	.712	.712	.450	.888	.894	.894	.648	.995	.987	.986	.574	.993	.984	.982	.673	.994	.983	.983
AMH drug class minor sartans	7	.995	1	1	1	.993	1	1	1	.995	1	1	1	.996	1	1	1	.996	1	1	1
AMH drug class minor sedating antihistamines	5	.911	.983	.940	.940	.946	.982	.906	.916	.942	.987	.966	.885	.860	.982	.931	.930	.863	.980	.857	.903
AMH drug class minor selective alpha blockers	2	.400	.668	.662	.661	.450	.765	.758	.758	.499	.983	.978	.976	.499	.988	.983	.980	.349	.692	.692	.691
AMH drug class minor selective alpha blockers genitourinary	3	.483	.532	.532	.532	.499	.662	.664	.664	.749	.865	.848	.848	.749	.865	.865	.864	.898	.996	.997	.996
AMH drug class minor selective oestrogen receptor modulators	2	.450	.683	.682	.682	.250	.494	.494	.493	.749	1	.999	.999	.750	.997	.996	.996	.625	.999	.996	.995
AMH drug class minor selective serotonin reuptake inhibitors	6	.828	.833	.833	.833	.826	.833	.833	.833	.993	1	.998	.965	.995	1	.999	1	.995	.999	.999	.999
AMH drug class minor sirolimus derivatives	2	.500	1	1	1	.250	.500	.500	.500	.500	1	1	1	.525	1	.999	1	.525	.999	.998	.998

(Continue on next page)

Table A3. Cross-validation results by algorithms and drug characteristics (cont'd)

		AUC (method / algorithm)																			
		cdf				*ctf*				*cdf-icdf*				Stemming				Drug synonyms			
Characteristic name	n	NB	IBk	SVL	SVR	NB	IBk	SVL	SVR	NB	IBk	SVL	SVR	NB	IBk	SVL	SVR	NB	IBk	SVL	SVR
AMH drug class minor snake antivenoms	7	.998	1	1	1	.996	1	.993	1	.997	1	.999	.971	.998	1	.986	1	.998	1	.978	.978
AMH drug class minor somatostatin analogues	2	.400	.800	.799	.800	.250	.441	.438	.439	.875	1	1	1	.875	1	1	1	.875	1	1	.999
AMH drug class minor statins	5	.997	1	1	1	.997	1	.970	.970	.997	1	1	1	.997	1	1	1	.997	1	1	1
AMH drug class minor stimulant laxatives	3	.998	1	.982	.999	.932	.999	.998	.999	.999	.999	1	1	.963	.998	.997	.997	.998	.999	.998	.998
AMH drug class minor stool softeners	3	.825	.886	.905	.856	.758	.760	.764	.764	.848	.982	.982	.984	.795	.913	.911	.918	.780	.954	.950	.956
AMH drug class minor substance p antagonists	2	.275	.500	.500	.500	.500	.998	.971	.996	.504	.534	.171	.171	.750	.714	.727	.727	.750	.766	1	1
AMH drug class minor sulfonylureas	4	.995	.998	.998	.998	.995	.998	.997	.999	.995	.998	.998	.998	.996	.998	.998	.999	.997	.999	.999	1
AMH drug class minor sympathomimetics anaphylaxis	1	-	-	-	-	-	-	-	-	-	-	-	-	-	-	-	-	-	-	-	-
AMH drug class minor sympathomimetics cardiovascular	4	.832	.885	.849	.824	.640	.667	.643	.644	.847	.940	.879	.874	.854	.954	.917	.948	.845	.952	.923	.901
AMH drug class minor tars	3	.340	.449	.449	.450	.773	.927	.971	.971	.743	.685	.551	.549	.653	.209	.610	.687	.791	.308	.729	.743
AMH drug class minor taxanes	2	.500	.997	.989	.988	0	0	0	0	.600	1	.998	.998	.625	1	.998	.998	.525	.998	.997	.997
AMH drug class minor tetracyclines	3	.999	.999	.998	.998	.997	.998	.965	.965	.982	.999	.998	.982	.999	.999	.965	.965	.999	.999	.931	.915
AMH drug class minor theophyllines	3	.532	.533	.533	.533	.843	.853	.853	.855	.665	.666	.666	.666	.665	.666	.665	.666	.666	.667	.665	.666
AMH drug class minor thiazide and related diuretics	3	.998	1	1	1	.999	1	.999	.999	.999	1	1	1	.999	1	1	1	.998	1	1	1
AMH drug class minor thiazolidinediones	2	.500	1	.998	.998	.500	.999	.996	.995	.925	1	1	1	.900	1	1	1	1	1	1	1
AMH drug class minor thrombolytics	5	.995	1	1	1	.994	1	1	1	.997	1	1	1	.996	1	1	1	.995	1	1	1
AMH drug class minor thyroid hormones	2	.525	.987	.988	.987	.250	.500	.500	.500	.525	1	1	1	.650	1	1	1	.599	.900	.899	.900
AMH drug class minor tnf alpha antagonists	3	.981	1	1	1	.999	1	1	1	1	1	1	1	1	1	1	1	.999	1	.999	1
AMH drug class minor topoisomerase i inhibitors	2	.500	.994	.989	.988	.200	.395	.393	.393	.599	.996	.996	.995	.525	.994	.996	.994	.524	.996	.994	.994
AMH drug class minor tricyclic antidepressants	7	.993	1	1	.999	.994	1	.956	.999	.995	.999	1	.999	.994	1	1	1	.994	1	.999	.999
AMH drug class minor triptans	3	.400	.400	.400	.400	.333	.333	.333	.333	.999	1	1	1	1	1	1	1	.999	1	1	1
AMH drug class minor tyrosine kinase inhibitors	8	.994	.999	1	1	.994	1	.997	.998	.997	.999	1	1	.994	1	.999	.999	.994	.999	.992	.993
AMH drug class minor vaccines	17	.998	1	1	1	.995	1	1	1	.997	1	1	1	.998	1	1	1	.999	1	1	1
AMH drug class minor vasoconstrictors eye	3	.595	.729	.727	.727	.905	.950	.922	.870	.530	.653	.589	.625	.579	.664	.661	.660	.579	.665	.581	.630
AMH drug class minor vertigo	1	-	-	-	-	-	-	-	-	-	-	-	-	-	-	-	-	-	-	-	-

(Continue on next page)

Table A3. Cross-validation results by algorithms and drug characteristics (cont'd)

		AUC (method / algorithm)																			
		cdf				*ctf*				*cdf-icdf*				Stemming				Drug synonyms			
Characteristic name	n	NB	IBk	SVL	SVR	NB	IBk	SVL	SVR	NB	IBk	SVL	SVR	NB	IBk	SVL	SVR	NB	IBk	SVL	SVR
AMH drug class minor vinca alkaloids	3	.277	.332	.332	.332	.578	.661	.660	.643	.912	.998	.997	.996	.928	.997	.997	.997	.894	.996	.997	.998
AMH drug class minor vitamin d	4	0	0	0	0	0	0	0	0	.743	.746	.748	.747	0	0	0	0	0	0	0	0
AMH drug class minor vitamin k antagonists	2	.500	.968	.962	.962	.500	.694	.694	.694	.500	.780	.779	.779	.500	.989	.978	.978	.450	.890	.887	.886
AMH drug class minor warts	4	.877	.901	.786	.823	.900	.920	.809	.813	.729	.879	.808	.774	.737	.710	.573	.571	.733	.854	.589	.599
AMH drug class musculoskeletal drugs	32	.953	.984	.986	.986	.954	.984	.985	.987	.977	.988	.991	.992	.973	.981	.990	.987	.970	.981	.988	.985
AMH drug class neurological drugs	59	.974	.977	.986	.984	.967	.967	.982	.975	.948	.960	.977	.976	.963	.966	.972	.964	.969	.971	.989	.985
AMH drug class obstetric and gynaecological drugs	37	.920	.918	.946	.938	.931	.904	.966	.961	.914	.955	.923	.913	.907	.908	.935	.916	.898	.914	.939	.905
AMH drug class psychotropic drugs	66	.966	.981	.981	.978	.964	.986	.987	.982	.970	.981	.970	.971	.962	.943	.966	.970	.972	.987	.982	.985
AMH drug class respiratory drugs	28	.884	.925	.893	.891	.873	.882	.896	.895	.931	.945	.913	.932	.925	.884	.936	.935	.939	.866	.954	.938
AMH drug class vaccines	17	.998	1	1	1	.994	1	1	1	.998	1	1	1	.998	1	1	1	.999	1	1	1
PKIS perpetrators CYP1A2 inducers	2	.500	.467	.467	.467	0	0	0	0	.449	.462	.429	.430	.399	.359	.359	.359	.299	.326	.323	.323
PKIS perpetrators CYP1A2 inhibitors	5	.582	.576	.585	.584	.714	.690	.493	.546	.588	.427	.591	.588	.602	.572	.646	.642	.535	.541	.543	.544
PKIS perpetrators CYP1A2 moderate inhibitors	2	.500	.995	.994	.993	.250	.498	.499	.497	.599	.999	.998	.998	.599	.998	.998	.997	.575	.999	.999	.999
PKIS perpetrators CYP1A2 strong inhibitors	3	.350	.455	.467	.456	.480	.543	.547	.539	.422	.247	.440	.440	.681	.729	.757	.741	.729	.755	.810	.796
PKIS perpetrators CYP2C19 inducers	3	.599	.805	.766	.794	.843	.972	.940	.956	.785	.929	.889	.874	.801	.965	.872	.842	.818	.966	.858	.859
PKIS perpetrators CYP2C19 inhibitors	6	.670	.916	.773	.830	.524	.946	.775	.897	.706	.833	.841	.835	.669	.855	.695	.822	.662	.862	.603	.825
PKIS perpetrators CYP2C19 moderate inhibitors	3	.531	.584	.524	.488	.179	.196	.195	.195	.518	.689	.505	.517	.564	.835	.590	.644	.643	.804	.609	.637
PKIS perpetrators CYP2C19 strong inhibitors	3	.616	.753	.560	.563	.380	.544	.426	.428	.646	.893	.667	.696	.525	.894	.696	.699	.539	.825	.682	.688
PKIS perpetrators CYP2D6 inhibitors	11	.844	.913	.742	.686	.939	.901	.347	.417	.868	.834	.581	.632	.885	.866	.793	.827	.769	.825	.800	.778
PKIS perpetrators CYP2D6 moderate inhibitors	8	.778	.853	.589	.583	.388	.853	.545	.699	.799	.722	.550	.563	.844	.783	.756	.736	.884	.765	.741	.653
PKIS perpetrators CYP2D6 strong inhibitors	3	.663	.664	.663	.663	.545	.592	.592	.590	.745	.794	.761	.777	.763	.796	.778	.778	.811	.860	.827	.827
PKIS perpetrators CYP3A4 inducers	5	.772	.780	.632	.735	.574	.756	.706	.800	.766	.740	.721	.713	.799	.826	.809	.792	.814	.854	.793	.812
PKIS perpetrators CYP3A4 inhibitors	21	.884	.897	.867	.909	.878	.910	.898	.911	.895	.872	.911	.906	.883	.857	.904	.895	.883	.849	.870	.895
PKIS perpetrators CYP3A4 moderate inhibitors	8	.629	.693	.514	.541	.714	.736	.680	.660	.679	.616	.458	.458	.457	.565	.448	.557	.576	.597	.402	.513
PKIS perpetrators CYP3A4 strong inhibitors	13	.905	.970	.969	.972	.915	.971	.926	.982	.953	.975	.985	.981	.935	.965	.969	.964	.944	.989	.987	.985

(Continue on next page)

Table A3. Cross-validation results by algorithms and drug characteristics (cont'd)

		AUC (method / algorithm)																			
		cdf				*ctf*				*cdf-icdf*				Stemming				Drug synonyms			
Characteristic name	n	NB	IBk	SVL	SVR	NB	IBk	SVL	SVR	NB	IBk	SVL	SVR	NB	IBk	SVL	SVR	NB	IBk	SVL	SVR
PKIS victim class alkylating agents	12	.911	.917	.905	.920	.990	.999	.994	.995	.991	.997	1	1	.989	.996	.990	.989	.989	.994	.999	.989
PKIS victim class anaesthetics	1	-	-	-	-	-	-	-	-	-	-	-	-	-	-	-	-	-	-	-	-
PKIS victim class anthracyclines	5	.793	.800	.800	.800	.850	.880	.880	.880	.992	.996	.967	.967	.991	.999	.998	.998	.992	.997	.998	.997
PKIS victim class antiarrythmics	4	.938	.989	.601	.765	.921	.952	.847	.852	.968	.993	.861	.864	.993	.996	.870	.968	.991	.995	.915	.895
PKIS victim class antibacterial	3	.736	.846	.844	.842	.609	.686	.685	.685	.826	.986	.956	.957	.887	.985	.937	.937	.903	.982	.904	.905
PKIS victim class antibodies	8	.984	.985	.893	.934	.979	.987	.865	.905	.978	.992	.972	.980	.981	.986	.977	.976	.983	.990	.965	.960
PKIS victim class anticonvulsants	12	.975	.997	.995	.997	.995	.999	.971	.997	.997	.996	.996	.995	.996	.998	.993	.998	.997	.996	.992	.996
PKIS victim class antipsychotics	2	.400	.709	.715	.739	.300	.528	.509	.506	.499	.918	.922	.737	.419	.570	.568	.568	.447	.661	.657	.657
PKIS victim class antithrombotic	9	.989	.999	.977	.999	.992	.999	.999	.999	.992	.999	.998	.998	.991	.999	.999	.999	.991	.999	.999	.999
PKIS victim class corticosteroids	6	.905	.965	.968	.931	.815	.929	.829	.932	.741	.741	.720	.729	.971	.971	.849	.901	.869	.656	.882	.881
PKIS victim class immunosuppressants	6	.908	.912	.877	.911	.222	.264	.248	.240	.948	.991	.953	.962	.949	.990	.970	.994	.948	.990	.961	.955
PKIS victim class oral hypoglycaemics	3	.879	.929	.927	.929	.879	.989	.994	.994	.980	.998	.999	.999	.880	.998	.997	.998	.963	.996	.996	.996
PKIS victim class sympathomimetic	1	-	-	-	-	-	-	-	-	-	-	-	-	-	-	-	-	-	-	-	-
PKIS victims list	72	.774	.778	.792	.784	.808	.836	.824	.826	.814	.825	.809	.805	.731	.776	.771	.783	.731	.769	.737	.734

(End of Table)
